# Quantifying Muscle Forces and Joint Loading During Hip Exercises Performed With and Without an Elastic Resistance Band

**DOI:** 10.3389/fspor.2021.695383

**Published:** 2021-08-23

**Authors:** Callum Buehler, Willi Koller, Florentina De Comtes, Hans Kainz

**Affiliations:** Neuromechanics Research Group, Department of Biomechanics, Kinesiology and Computer Science in Sport, Centre for Sport Science and University Sports, University of Vienna, Vienna, Austria

**Keywords:** elastic resistance band, musculoskeletal simulations, hip joint contact force, muscle force, hip strengthening exercises, OpenSim, rehabilitation

## Abstract

An increase in hip joint contact forces (HJCFs) is one of the main contributing mechanical causes of hip joint pathologies, such as hip osteoarthritis, and its progression. The strengthening of the surrounding muscles of the joint is a way to increase joint stability, which results in the reduction of HJCF. Most of the exercise recommendations are based on expert opinions instead of evidence-based facts. This study aimed to quantify muscle forces and joint loading during rehabilitative exercises using an elastic resistance band (ERB). Hip exercise movements of 16 healthy volunteers were recorded using a three-dimensional motion capture system and two force plates. All exercises were performed without and with an ERB and two execution velocities. Hip joint kinematics, kinetics, muscle forces, and HJCF were calculated based on the musculoskeletal simulations in OpenSim. Time-normalized waveforms of the different exercise modalities were compared with each other and with reference values found during walking. The results showed that training with an ERB increases both target muscle forces and HJCF. Furthermore, the ERB reduced the hip joint range of motion during the exercises. The type of ERB used (soft vs. stiff ERB) and the execution velocity of the exercise had a minor impact on the peak muscle forces and HJCF. The velocity of exercise execution, however, had an influence on the total required muscle force. Performing hip exercises without an ERB resulted in similar or lower peak HJCF and lower muscle forces than those found during walking. Adding an ERB during hip exercises increased the peak muscle and HJCF but the values remained below those found during walking. Our workflow and findings can be used in conjunction with future studies to support exercise design.

## Introduction

Persistent symptomatic problems of the hip joint have been shown to cause a substantial impact on the overall health in the older population (Dawson et al., 2005). This is especially problematic considering that one in five people aged 65 years and older experience hip pain (Dawson et al., 2004). Some of the conditions that cause this hip pain, such as osteoarthritis (OA), have no cure and can cause an accelerated progression, leading to a high rate of surgical interventions (Gossec et al., 2005). Joint degeneration in the hip and knee OA is associated with altered gait patterns (Astephen et al., 2008; Eitzen et al., 2012; Meyer et al., 2015, 2018). These altered gait patterns often lead to joint pathomechanics such as high joint contact forces, which accelerate the progression of the disease (Meireles et al., 2017; Richards et al., 2018).

Compensatory movement strategies found in patients with hip OA are often a result of the observed hip muscle weakness (Meyer et al., 2018). A systemic review by Loureiro et al. (2013) highlighted that the affected legs of hip OA show significantly lower muscle strength compared to both the contralateral leg and/or healthy controls. Strengthening the joint supporting muscles is used as a conservative treatment to improve the quality of life of patients and to slow down the progression of OA (Zhang et al., 2008; Nho et al., 2013). The required muscle stimulus for muscle strengthening can be achieved with different exercise modalities (Hofmann et al., 2016; Iversen et al., 2018).

For muscle-strengthening exercises, elastic resistance bands (ERBs) are especially an easy-to-use, cheap, and effective alternative to conventional resistance-training equipment (Cambridge et al., 2012; Sundstrup et al., 2014; Calatayud et al., 2015; Aboodarda et al., 2016). Previous studies investigated the material properties of ERBs (Simoneau et al., 2001; Santos et al., 2009; Uchida et al., 2016). These studies highlighted that the resistance force increases linearly with the elongation of the ERB. Furthermore, the force–elongation characteristics differ between ERBs with different stiffnesses. Due to this predictive, linear behavior, as well as to the other benefits mentioned above, ERBs present an ideal and practical training method for rehabilitation exercises. However, to the best of the knowledge of the authors, no studies assessed the impact of ERBs on muscle and joint contact forces.

Strengthening the hip muscles increases the stability of the joint and reduces joint contact forces (Retchford et al., 2013; Meyer et al., 2018). In other words, increased stability due to a more balanced muscle force distribution reduces femoral head translation and therefore decreases joint contact forces. This is especially critical because the presence of increased hip joint contact forces (HJCFs) is one of the main contributing mechanical causes of hip OA and its progression (Recnik et al., 2009; Felson, 2013). Therefore, the knowledge, understanding, and subsequent control of these forces are essential for building a progressive rehabilitation program. Despite the link between muscle weakness, joint contact forces, and OA progression, recommendations for rehabilitative muscle-strengthening exercises are often based on an expert opinion instead of the supporting scientific evidence (Conaghan et al., 2008; Zhang et al., 2008).

Only a small number of studies investigated the impact of hip exercises on HJCF. While a plethora of literature on the relationship between HJCF and movements, such as walking, running, and stair climbing exist (Heller et al., 2001; Bergmann et al., 2004; Lenaerts et al., 2008; Giarmatzis et al., 2015, 2017; Wesseling et al., 2015; Meyer et al., 2018; Kainz et al., 2020), only sporadic research has been done regarding other activities, such as single-leg standing or cycling (Bergmann et al., 2001; Varady et al., 2015; Damm et al., 2017) and even less that have dealt with specific hip-strengthening exercises. Catelli et al. (2020) compared HJCFs during a squat between patients with the cam-type femoroacetabular impingement for both pre- and post-hip-corrective surgeries and with those of a healthy control in which they found no significant difference. *In vivo* measurements *via* instrumented endoprosthesis showed that only weight-bearing exercises caused significantly high HJCF (up to 441% of the body weight), whereas most of the others, such as non-weight-bearing, isometric exercises, did not (Schwachmeyer et al., 2013). Investigation on the impact of alternative weight-bearing training modalities, such as ERB exercises, on HJCF, is still missing.

The goal of this study was to (1) quantify the muscle forces and the accompanying loading on the hip joint during ERB exercises, which target muscles shown to promote joint stability, and (2) compare these forces to those observed during walking. Our participants performed hip-strengthening exercises with two different ERBs and execution velocities. During all exercises, the participants were standing on one leg and performed the movement with the contralateral leg. We hypothesized that (1) muscle forces and HJCF are higher when using a stiffer ERB compared with those using a softer ERB and no ERB, (2) movement execution with a higher velocity will increase the peak HJCF but decrease the total muscle forces, and (3) the peak and total muscle forces but not the peak HJCF of the movement leg will be higher compared with walking. In addition, we analyzed and compared joint kinematics, joint kinetics, and ERB forces between the different exercise modalities, i.e., different ERB and execution velocities, to get a comprehensive overview of the impact of ERB exercises on the musculoskeletal system.

## Materials and Methods

Three-dimensional motion capture data and ground reaction forces were collected during the typical hip muscle-strengthening exercises used in the rehabilitation of hip pathologies. These data were used for musculoskeletal simulations to estimate the muscle forces and HJCF.

### Participants

Sixteen healthy adults (11 men and 5 women) with no pre-existing or acute lower limb pathologies were recruited *via* word of mouth and participated in our study. Their average ± SD age, weight, height, and body mass index were 27 ± 4 years, 70.7 ± 12.5 kg, 1.75 ± 0.10 m, and 22.9 ± 2.8 kg m^−2^, respectively. The research ethics and methods of the study were approved by the Ethics Committee of the University of Vienna (00579), and all participants were informed regarding the purpose of the study and gave their written consent before participation.

### Exercises

All participants performed rehabilitation exercises that aimed to strengthen the following hip-stabilizing muscles: (a) hip abductors including gluteus medius, gluteus minimus, tensore facie latae, and piriformis (Valente et al., 2013; Meyer et al., 2018); (b) hip flexors including rectus femoris, iliacus, psoas, iliocapsularis, and sartorius (Zhang et al., 2008); and (c) hip extensors including gluteus maximus, biceps femoris, semitendinosus, and semimembranosus (Loureiro et al., 2013). The exercises were performed in a standing straight-legged position, and each muscle group was targeted with a separate exercise. All exercises were first performed without the use of an ERB and then subsequently with two different elastic band types that differed in their resistance-elongation characteristics. The order of the used ERB was the same for each participant.

All exercises were performed at a slow executing speed and a fast executing speed. A metronome was used to standardize the execution velocity. Participants were instructed to start the movement with a beat, to be at the end of their range of motion by the following beat, and to be back in the initial starting position by the third beat. Using 40 beats per minute for the slow and 60 beats per minute for the fast variant resulted in an exercise duration of 3 and 2 s for the slow and fast movement execution, respectively.

**Figure 1:**
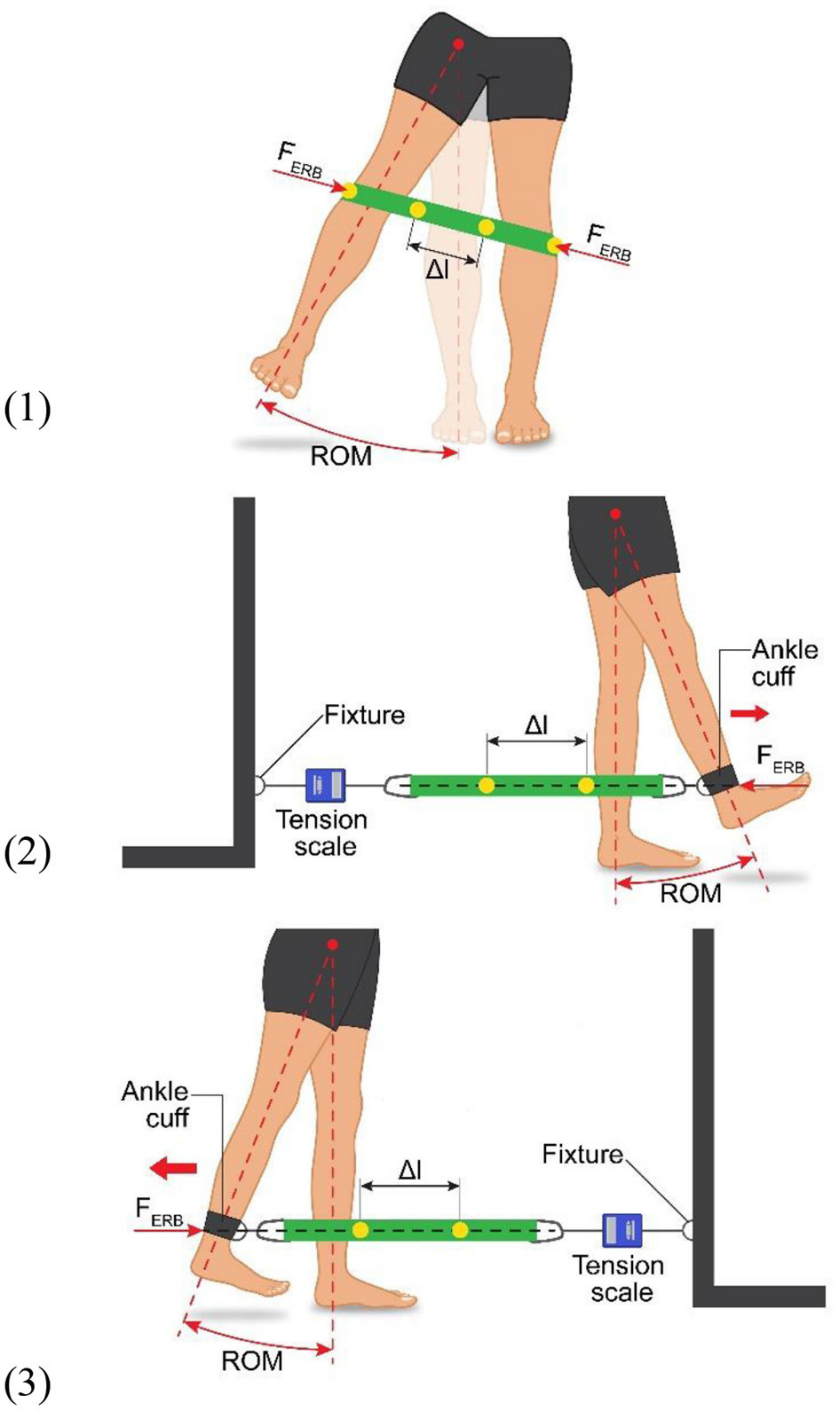
Experimental setup showing the movement execution, as well as showing the elastic resistance band (ERB) fastening method and position for all three exercises: (1) standing single-leg abduction, (2) standing single-leg hip extension, and (3) standing single-leg hip flexion. The dimension Δ*l* shows the measured marker displacement used to calculate the force production of the ERB (i.e., F_ERB_).

Each participant performed five gait trials by walking over a 10 m long designated runway with embedded force plates at a self-selected walking speed. Each gait trial was cropped to one gait cycle. Subsequently, each participant executed the following three exercises: (1) a standing single-leg abduction, (2) a standing single-leg hip extension, and (3) a standing single-leg hip flexion (Figure 1). During all exercise trials, the participants were instructed to keep their hands on their hips and to look straight ahead and use their full range of motion while keeping their torso as still and upright as possible. During the initial position of all exercises, the participants were standing with each foot on one force plate. To standardize the foot position for each participant, the distance between the left and right anterior superior iliac spine anatomical landmarks was measured and marked on the floor prior to the start of the trials. The participants were asked to place their heels on the markings and point their toes forward, to ensure both feet were parallel to each other. Furthermore, the participants were asked to keep their knees straight and to exert a slight dorsiflexion with the foot during the entire course of the movements. For each exercise and condition (Table 1), at least five trials were collected for each condition. Each participant was instructed to perform the exercises at a rate of the perceived exertion of 4 out of 10, which corresponds to a contraction intensity of ~40% of the maximum voluntary contraction or a training intensity level of a warm-up (Morishita et al., 2013).

**Table 1:** Exercise variations and conditions.

**Exercise**	**Condition**	**Velocity**
No exercise	Walking	Self-selected speed
Hip abduction	No ERB	Slow (3 s)
		Fast (2 s)
	Softer ERB	Slow (3 s)
		Fast (2 s)
	StifferERB	Slow (3 s)
		Fast (2 s)
Hip flexion	No ERB	Slow (3 s)
		Fast (2 s)
	SofterERB	Slow (3 s)
		Fast (2 s)
	StifferERB	Slow (3 s)
		Fast (2 s)
Hip extension	No ERB	Slow (3 s)
		Fast (2 s)
	SofterERB	Slow (3 s)
		Fast (2 s)
	StifferERB	Slow (3 s)
		Fast (2 s)

For the ERB trials, the bands were secured in place to ensure that they would not move during the exercises. For the flexion and extension trials, a fixture was used that was aligned with the movement leg (Figure 1). An ankle cuff was used to attach the ERB to the leg, while the other end of the ERB was attached to the fixture. Furthermore, a tension scale was inserted between the fixture and the resistance band to standardize the band tension at the beginning of each exercise. A starting tension of 1 kg (9.81 N), with a tolerance of ±0.1 kg (0.98 N), was chosen. The cuff was placed above the ankle and was allowed to sit on the lateral and medial malleoli. To ensure a horizontal alignment of the ERB, the distance between the floor and the ankle cuff joint was measured and the joint on the opposite side between the fixture and the ERB was adjusted to match. The ERB, as well as the ankle cuff and fixture, was fitted with markers. To track the ERB elongation, two markers were placed on the ERB, each +10 cm and −10 cm from the midpoint of the loops, respectively. The two markers placed on the ankle cuff and the fixture were placed on the two lateral ERB loop apices during an abduction. These markers were subsequently used to define the force application point of the ERB.

### Three-Dimensional Motion Capture

To capture the movement of our participants, 21 retroreflective surface markers (Table 2) and 5 trilateral marker clusters were attached to the lower body and torso of each participant. In addition, four markers were used to track the ERB elongation. The subsequent marker trajectories were captured using a 12-camera optoelectronic system (Vicon Motion Systems, Oxford, UK) at a sampling frequency of 100 Hz. Simultaneously, synchronized ground reaction forces were collected *via* two embedded force plates (Kistler Instrumente AG, Switzerland) at a sample rate of 1,000 Hz. After collection, the marker trajectories were labeled, filtered, and cropped using Nexus 2.11.0 (Vicon Motion Systems, Oxford, UK).

**Table 2:** Marker set used for collecting the movement of our participants.

**Segment**	**Cluster**	**Marker**	**Placement**
Torso		C7	The Spinous process of the 7th cervical vertebra
		T10	The Spinous process of the 10th thoracic vertebra
		Clav	Centered between articuli sterno-clavicularis
		Sternum	Xiphoid process of the sternum
		Right_Back	Infraspinatus^b^
Pelvis	Pelvis cluster	PCL_CRAN	Resulting center of gravity of the isosceles triangle on center between left and right posterior superior iliac spine
		PCL_RECHTS	
		PCL_LINKS	
		RASI/LASI^a^	Right/left anterior superior iliac spine
Thigh	Right/left upper leg cluster	R/LCL_UL_CRAN	Mid-point between trochanter major and epicondyles lateralis^b^
		R/LCL_UL_POST	
		R/LCL_UL_ANT	
		Right/Left_Knee_LAT^a^	Epicondyles lateralis
		Right/Left_Knee_MED^a^	Epicondyles medialis
Lower leg	Right/left lower leg cluster	R/LCL_LL_CRAN	Mid-point between epicondyles lateralis and malleolus lateralis^b^
		R/LCL_LL_POST	
		R/LCL_LL_ANT	
		Right/Left_Ankle_LAT^a^	Malleolus lateralis
		Right/Left_Ankle_MED^a^	Malleolus medialis
Foot		Right/Left_Heel	Heel leveled with Right/Left_Toe
		Right/Left_Toe	2nd proximal interphalangeal joint
		Right/Left_M5	5th metatarsal head
Elastic resistance band		Inside_R/L	+10 cm/−10 cm resp. from band mid-point
		Outside_R/L	Lateral ERB apex during abduction, cuff and fix-point during flexion and extension

a *Remove after static calibration (for calibration purposes only)*.

b *Precise positioning not necessary*.

*The gray-shaded markers highlight cluster markers*.

### Elastic Resistance Band

**Figure 2:**
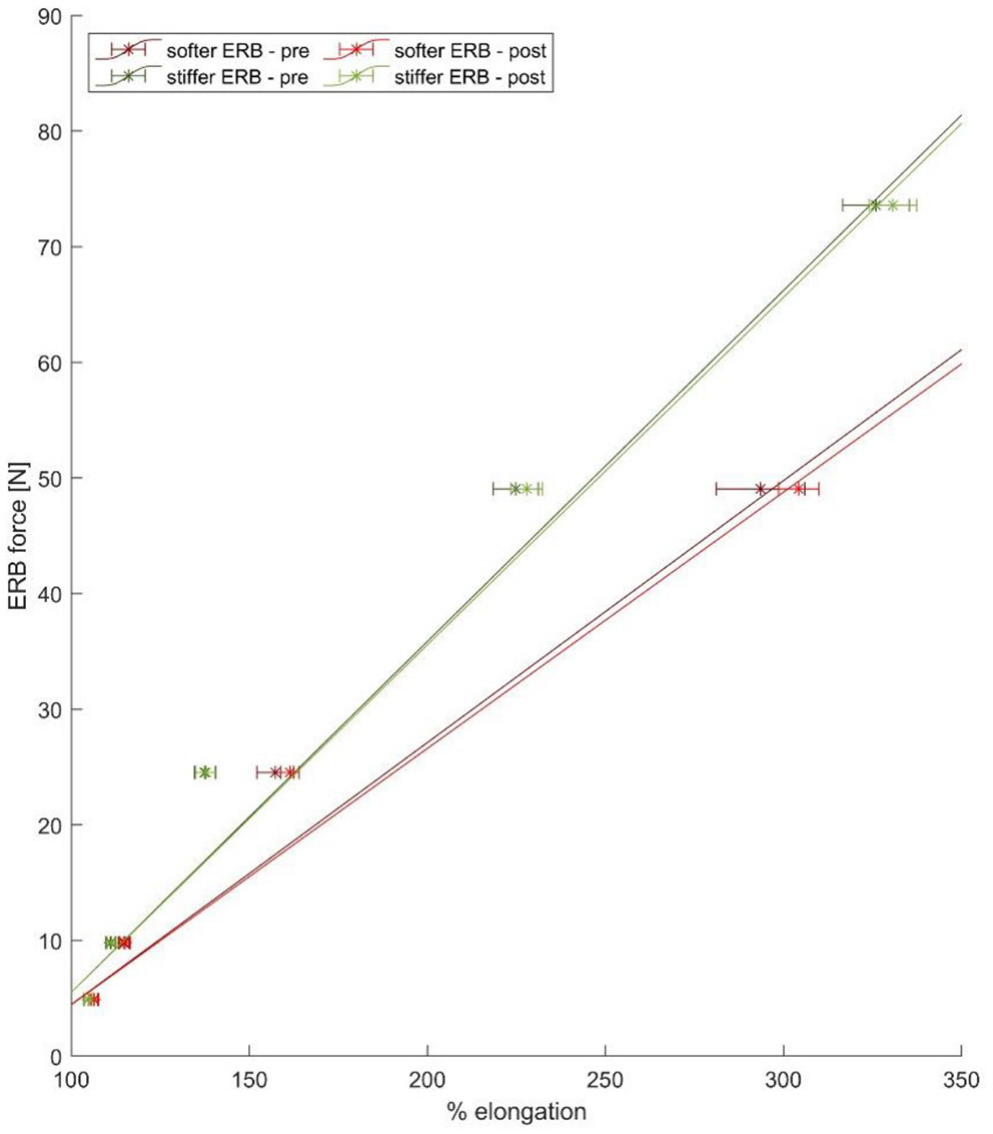
The mean force–elongation curve of the stiffer (green line) and softer (red line) ERBs obtained from the experimental data points (six points for the stiffer and five points for the softer ERB) based on the pre- (blue lines) and post (red lines)-data collection validation experiments.

Two ERBs of the brand Theraband (Thera-Band, OH, USA) were used in this study. The green ERB was the stiffer one, whereas the red ERB was the softer one. These two ERBs were chosen because they are often recommended by physiotherapists for home exercises. To ensure that the ratio of displacement to force production was consistent between the participants, as well as to validate the assumption of a linear relationship between the force and elongation, both ERBs used were evaluated before and after performing all trials of each participant. To verify the aforementioned assumptions, a series of different weights were affixed to the ERB and the elongation was measured using the Vicon system. The stiffer ERB was loaded with 0, 0.5, 1.0, 2.5, 5, and 7.5 kg, and the softer ERB was loaded with 0.0, 0.5, 1.0, 2.5, and 5 kg. The displacement of the attached reflective markers was measured and was subsequently used to fit a line to the experimental force, elongation data (Figure 2). The equation of the fitted line based on the ERB tests before the dynamic data collection with each participant was used to create the external force file for the dynamic musculoskeletal simulations (described in detail below). A paired *t*-test indicated no significant differences (*p* > 0.05) between the recorded elongation and the obtained fitted lines before and after the collection of the dynamic trial.

### Musculoskeletal Simulations

The generic “gait2392” OpenSim model (Delp et al., 1990) was scaled to the anthropometry of each participant using surface marker locations at anatomical landmarks and joint centers (Kainz et al., 2017). Due to insufficient markers at the foot, the metatarsophalangeal joints of the models were locked. The maximum isometric muscle forces were scaled depending on the body mass of the participants by Equation (1) (van der Krogt et al., 2016; Kainz et al., 2018).

(1) $$\begin{array}{l} F_{scaled}=\;F_{generic}\times {\left(\frac{m_{scaled}}{m_{generic}}\right)}^{\frac{2}{3}} \end{array}$$

The models of the participants and the corresponding motion capture data were used to run inverse kinematics followed by inverse dynamics, static optimization by minimizing the sum of squared muscle activations, and joint reaction load analyses with MATLAB R2020a (Mathworks Inc., Natick, MA, USA) and OpenSim 4.1 (Seth et al., 2018). The external force file used during the OpenSim simulations included the ground reaction forces from the force plates and the ERB forces obtained from the elongation of the ERB and the force–elongation curves. During inverse kinematic, all markers close to joint axes were excluded and only the cluster markers were tracked. Detailed information about which markers were included and their weighting factors can be found in the Supplementary Material. All scaling errors, as well as simulation errors, were below the best practice recommendations of OpenSim (Hicks et al., 2015).

### Validation of Simulations

To validate our simulation results, a qualitative visual comparison of the HJCF measured during each exercise was made with those found on OrthoLoad (Bergmann, 2008), a public database of HJCF measured *in vivo* with instrumented hip implants. The HJCF from all exercises in this study showed a reasonable agreement with the values from OrthoLoad (details can be found in the Supplementary Material).

### Data Processing and Statistical Analysis

For all analyzed parameters, the average waveform from approximately five trials per condition and participant was calculated and time-normalized. Gait trials were normalized to one gait cycle, whereas exercises were normalized to the movement cycle using the force plate data of the movement leg (movement started and ended when the foot left and hit the force plate, respectively). Furthermore, muscle forces and HJCFs were normalized to the body weight of each participant. For our first hypothesis, muscle force and HJCF waveforms were compared between exercises without and with ERBs. For each exercise, only the muscle group of interest was compared between the different conditions (e.g., average hip adductor muscle forces for the hip adductor exercise). For our second hypothesis, the peak HJCF and the force–time integral (FTI) were determined for each condition and compared between the slow and fast exercise executions. The FTI was used to estimate the total amount of muscle force needed for each exercise. We calculated the FTI by integrating the force of the corresponding muscle group over time, e.g., FTI for the hip adductor exercise was calculated by integrating the hip adductor muscle forces over time (Beltman et al., 2004; Ortega et al., 2015). For our third hypothesis, the peak HJCF, FTI, and peak muscle forces of the respective muscle groups of each exercise were compared with the same muscle groups during walking. Statistical parametric mapping (Pataky, 2010) based on the SPM1D package for Matlab (http://www.spm1d.org/) was used to statistically compare the waveforms for our first hypothesis. Within the SPM1D package, two-tailed scalar trajectory *t*-tests (SPM{t}) with Bonferroni adjusted alpha level (i.e., *p* = 0.05/3 = 0.0167 for the following comparisons: no ERB vs. softer ERB; no ERB vs. stiffer ERB; and softer vs. stiffer ERB) were chosen to compare the muscle forces and HJCF waveforms between exercises with and without ERB. IBM SPSS Statistics, version 27.0. (IBM, New York, USA) using repeated-measures ANOVA with a set significance level of *p* < 0.05 was used to compare the discrete parameters for our second and third hypotheses. For the second hypothesis, we used repeated measures ANOVA with the factors “ERB” (no ERB, softer ERB, stiffer ERB) and “speed” (fast, slow), whereas for our third hypothesis, we used repeated measures ANOVA with the factor “movement” (gait, exercise without ERB, exercise with softer ERB, exercise with stiffer ERB) and contrast-coded *post-hoc* tests (gait vs. all exercises) in case that the ANOVA revealed significant group differences. Repeated-measure results were verified with Greenhouse–Geisser corrections where the Mauchly test of sphericity determined the heterogeneity of covariance. In case of significant main effect, pairwise *post-hoc* comparison using Bonferroni-adjusted alpha levels was conducted. In addition, we assessed if there was a significant interaction between ERB and speed.

## Results

All the following figures, tables, and subsequent results presented pertain to the movement leg. The figures and graphs displaying the results of the standing leg and detailed statistical results (i.e., exact *p*-value for each comparison, *F* scores, partial eta-squared) can be found in the Supplementary Material of this study.

### Study Performance

While 16 participants performed the experiments, at various points in the data processing, some trials were either unusable or missing, e.g., missing markers, isolated muscle EMG signals unusable, or not all movement conditions performed. If this was the case, the incomplete or distorted data for the specific trial were discarded. However, this only applied to the isolated trial of the specific parameter. The total number of participants used in the final analysis is shown as “N” in Supplementary Tables 1, 2.

### Hypothesis 1: Muscle Forces and HJCF Are Higher When Using a Stiffer ERB Compared to Those Using a Softer ERB and No ERB

**Figure 3:**
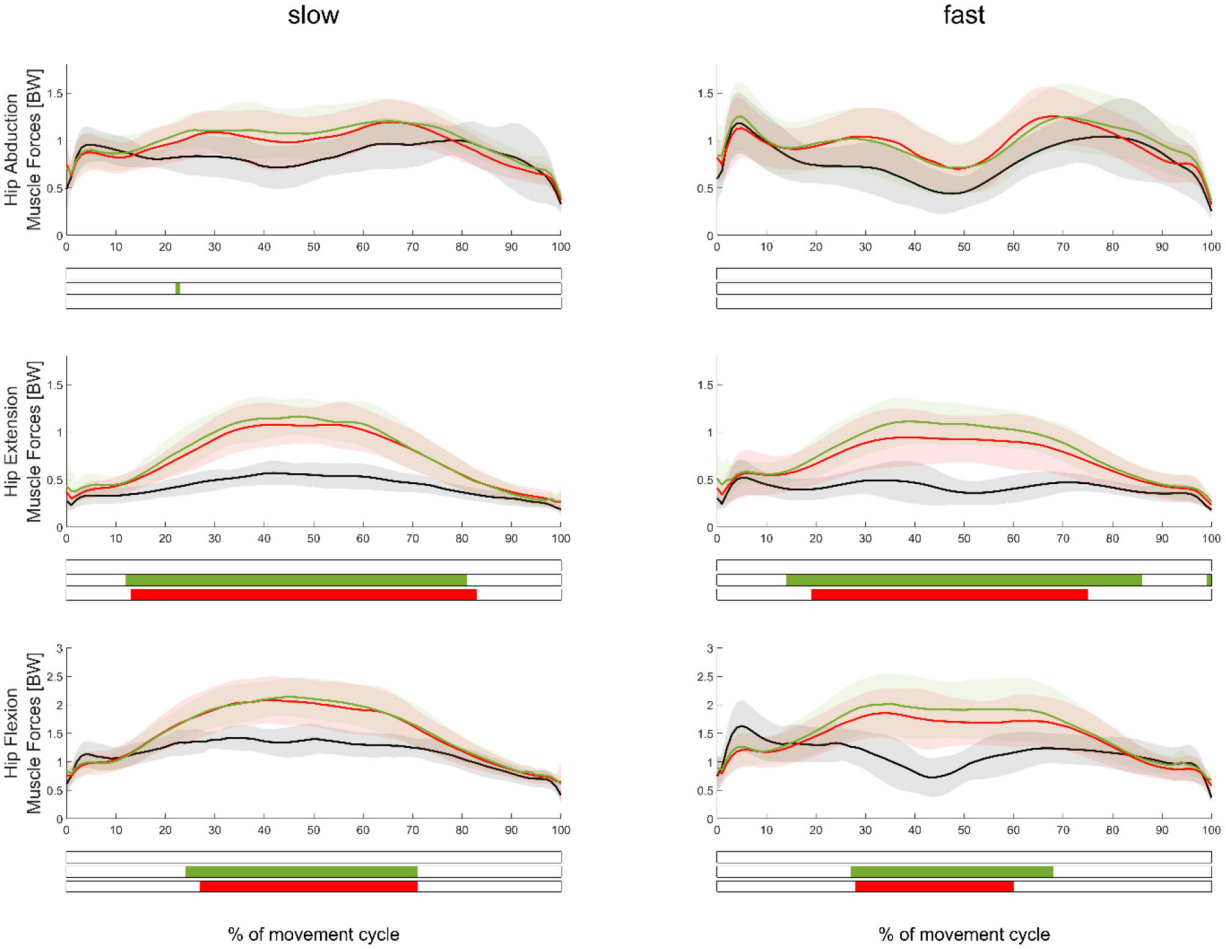
Mean (±SD) muscle force waveforms measured in the movement leg during hip abduction (top), extension (middle), and flexion (bottom) exercises, as well as during slow (left subplots) and fast (right subplots) velocities. Green, red, and black waveforms represent the stiffer, softer, and no ERB, respectively. Colored bars beneath each plot indicate significant differences between waveforms, whereas the green, red, and blue (first) bars represent significant differences between the stiffer vs. no ERB, softer vs. no ERB, and stiffer vs. softer ERB, respectively.

**Figure 4:**
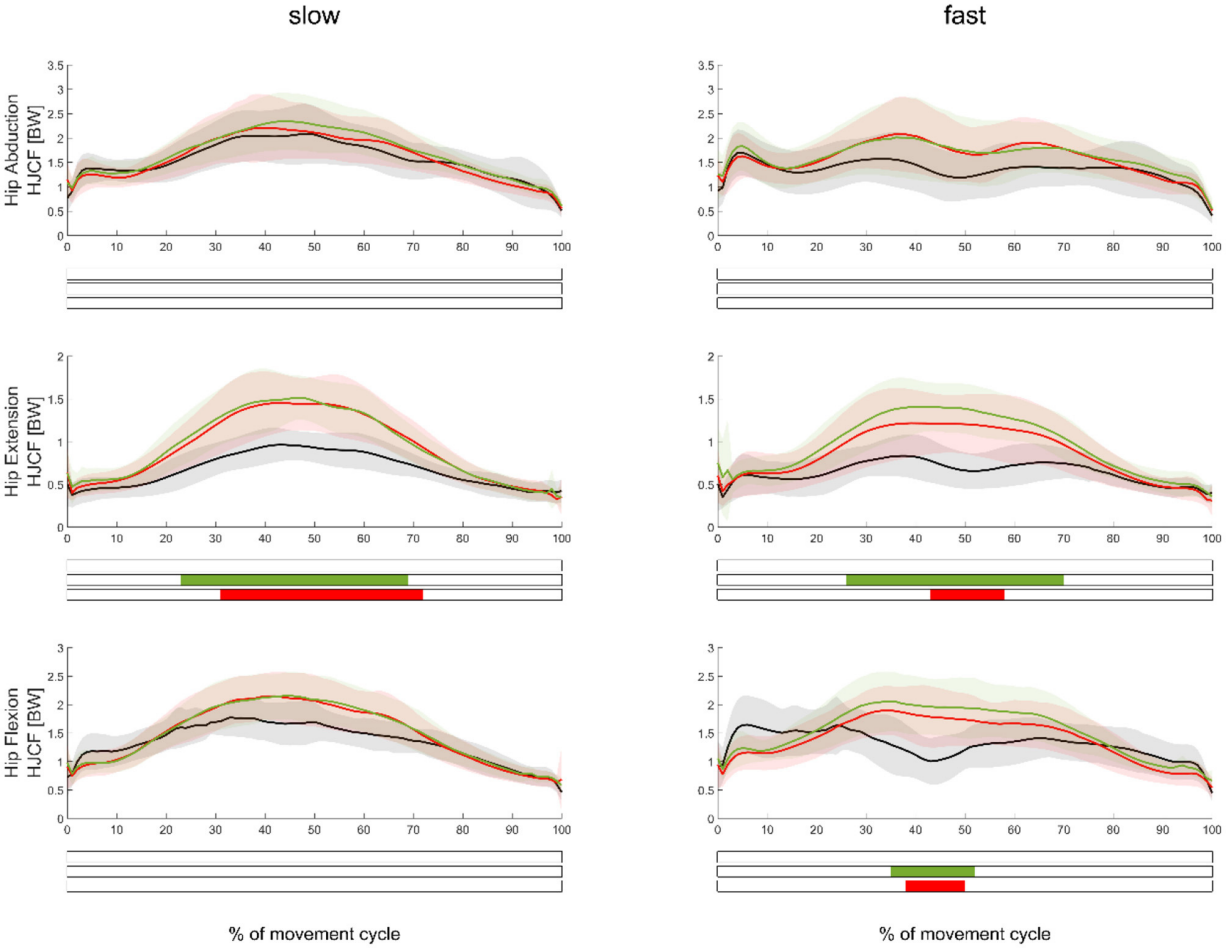
Mean (±SD) HJCF waveforms measured in the movement leg during hip abduction (top), extension (middle), and flexion (bottom) exercises, as well as during slow (left subplots) and fast (right subplots) velocities. Green, red, and black waveforms represent the stiffer, softer, and no ERB, respectively. Colored bars beneath each plot indicate significant differences between waveforms, whereas the green, red, and blue (first) bars represent significant differences between the stiffer vs. no ERB, softer vs. no ERB, and stiffer vs. softer ERB, respectively.

In regard to our first hypothesis, we found significantly higher (*p* < 0.0167) muscle forces during the middle part of the movement cycle when using an ERB (soft or stiff) compared to those using no ERB for hip extension and flexion exercises (Figure 3). HJCFs were significantly higher (*p* < 0.0167) during the middle part of the movement cycle when using an ERB (soft or stiff) compared to that using no ERB for hip extension exercises (fast and slow) and the fast hip flexion exercises (Figure 4). Performing the hip exercise with a stiffer or softer ERB did not show any significant differences in muscle forces and HJCFs.

**Figure 5:**
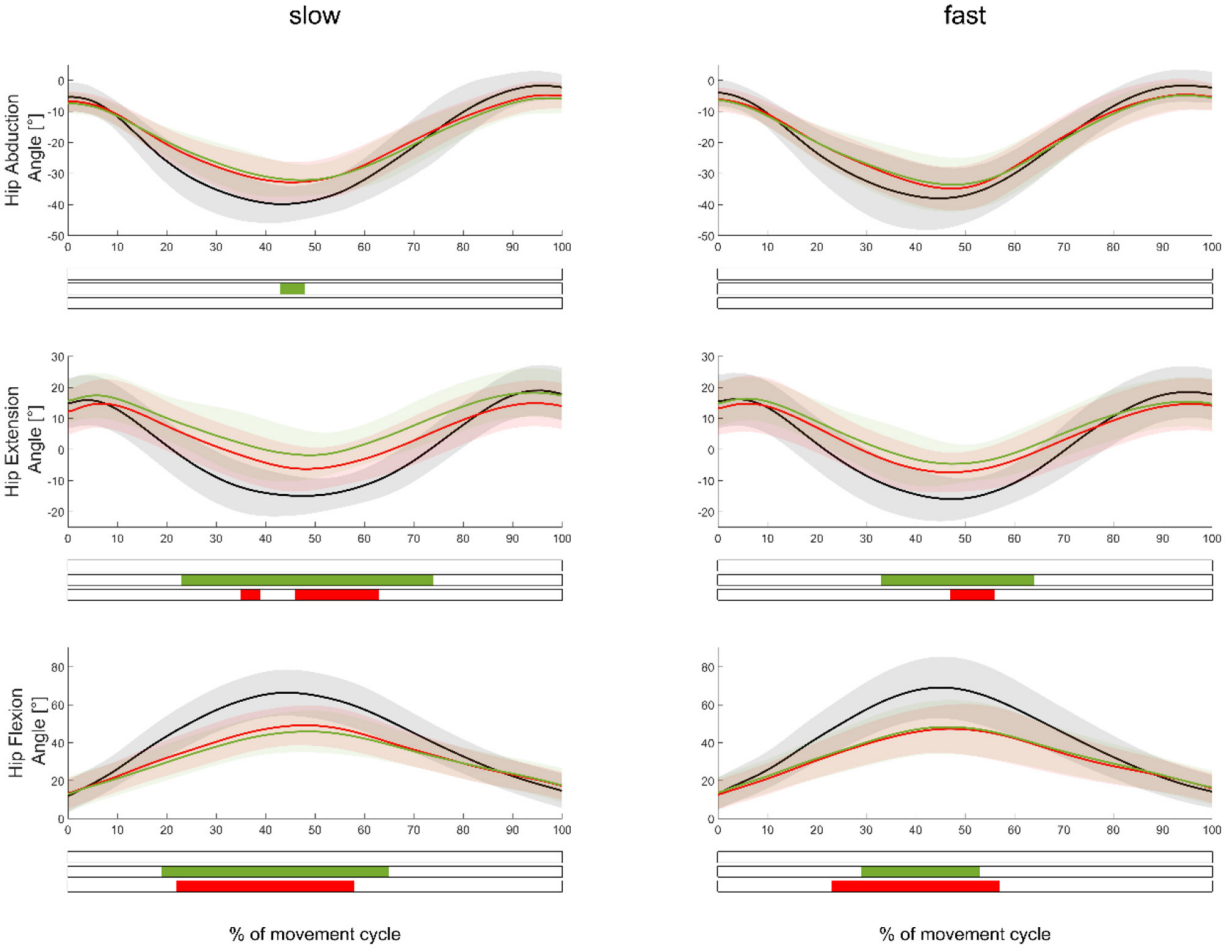
Mean (±SD) hip angle waveforms measured in the movement leg during hip abduction (top), extension (middle), and flexion (bottom) exercises, as well as during slow (left subplots) and fast (right subplots) velocities. Green, red, and black waveforms represent the stiffer, softer, and no ERBs, respectively. Colored bars beneath each plot indicate significant differences between waveforms, whereas the green, red, and blue (first) bars represent significant differences between the stiffer vs. no ERB, softer vs. no ERB, and stiffer vs. softer ERB, respectively.

**Figure 6:**
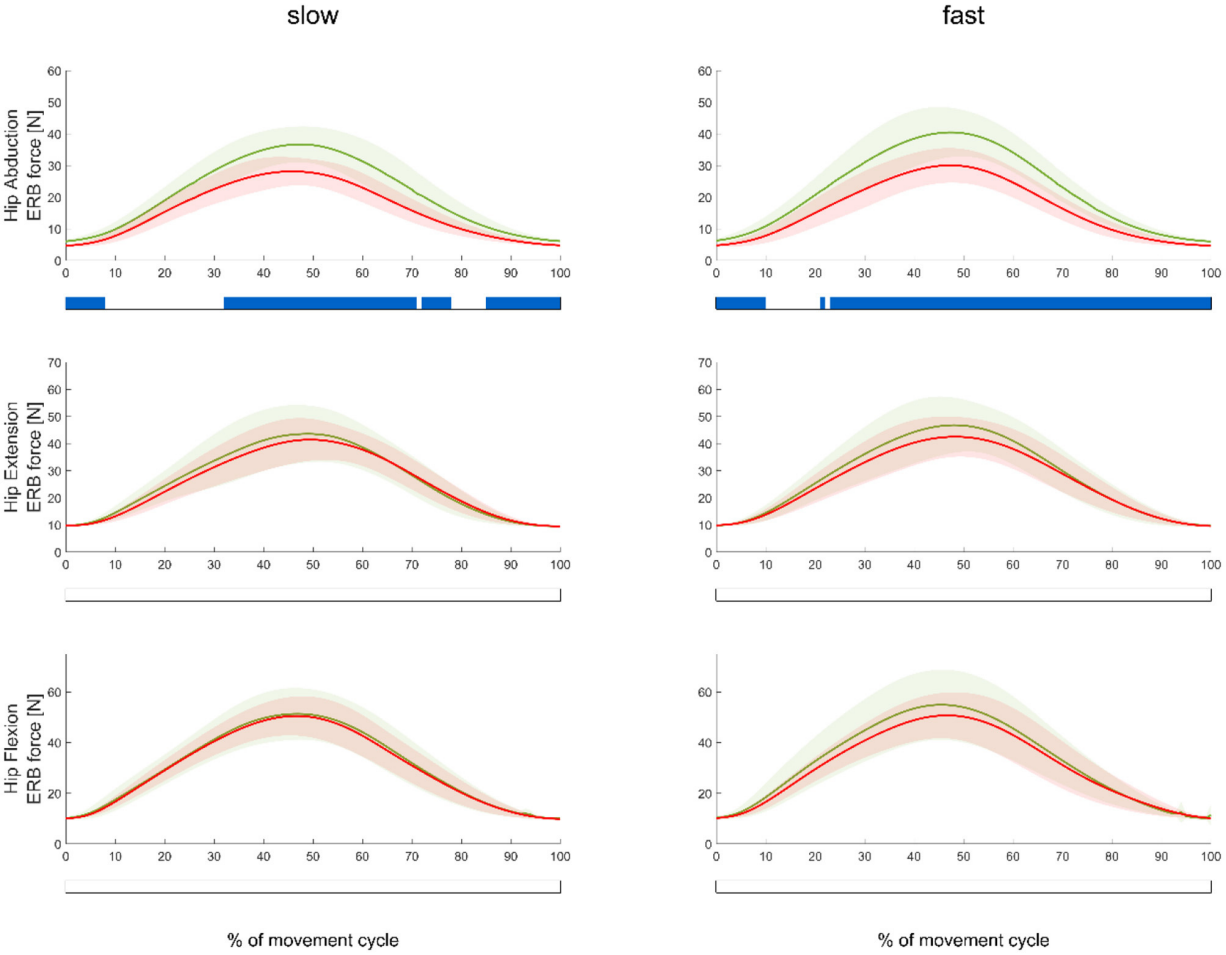
Mean (±SD) ERB forces during slow (left) and fast trials (right) measured in the softer (red waveform) and stiffer (green waveform) ERBs during abduction (top), extension (middle), and flexion (bottom) exercises. Blue bars beneath each plot indicate significant differences between the forces of the softer and stiffer ERBs.

The comparison of joint kinematics between the exercise execution variations without ERB and those with softer and stiffer ERBs showed several significant differences (Figure 5). The use of an ERB significantly decreased (*p* < 0.0167) the range of motion for hip extension and flexion exercises. Joint kinematics between exercises performed with the softer and stiffer ERBs were not significantly different. Similar to our muscle force results, joint moments of hip flexion and extension exercises were significantly higher (*p* < 0.0167) during the middle of the movement cycle when using an ERB compared with the exercise without the ERB (Supplementary Figure 2). ERB forces were only significantly higher when using the stiffer compared with those using the softer ERB (Figure 6) during hip abduction exercises.

### Hypothesis 2: Movement Execution With a Higher Velocity Will Increase the Peak HJCF but Decrease the Total Muscle Forces (FTI)

**Figure 7:**
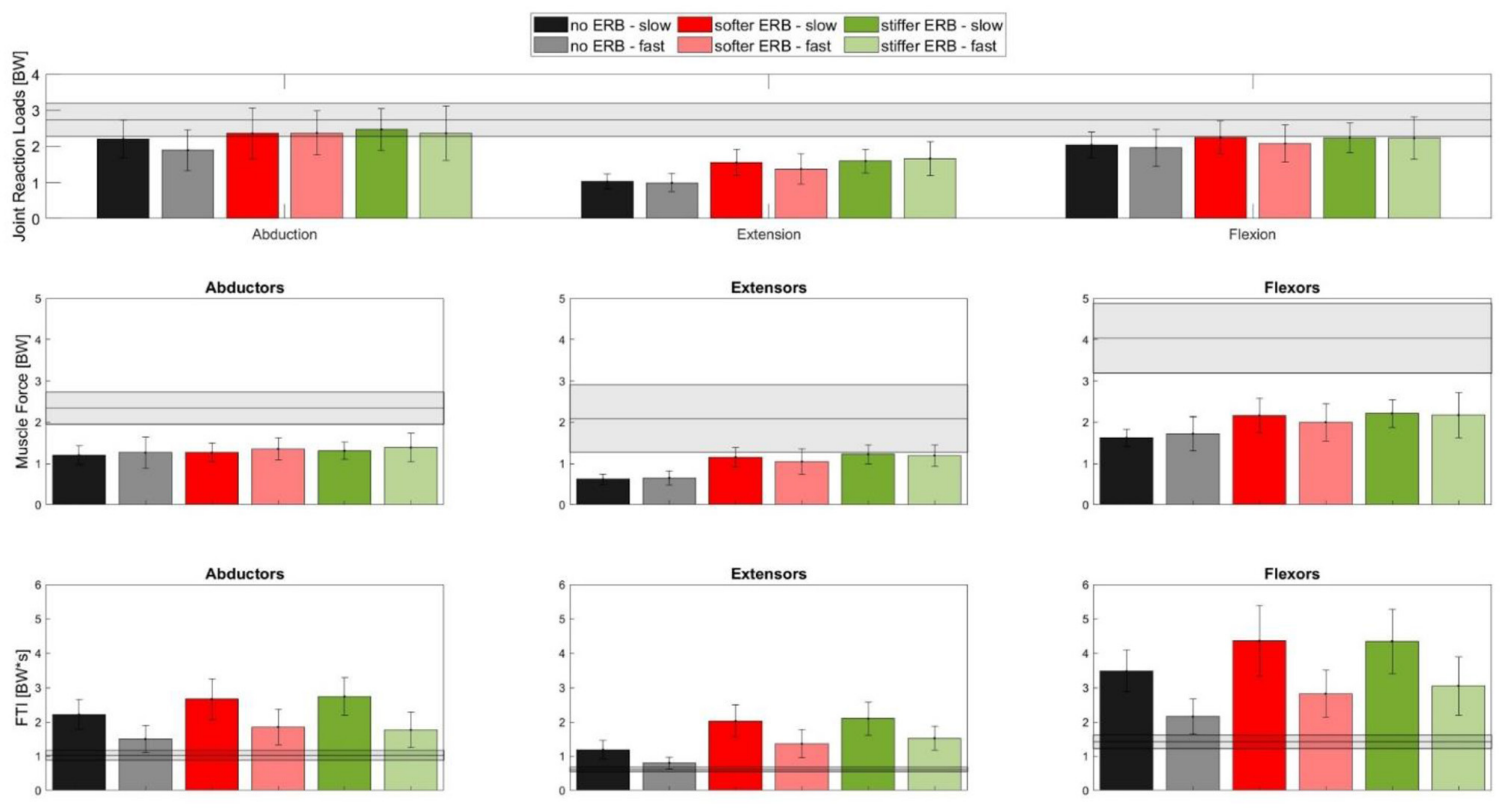
Bar plots showing the mean (±SD) of the peak HJCF (top row) during abduction (left), extension (middle), and flexion (right) as well as the peak muscle forces (middle row) and FTI (bottom row) of the respective target muscle groups (abductors, extensors, and flexors, shown in the left, middle, and right sides, respectively) in the movement leg. Each bar represents one of the execution variants (see the legend above). The gray horizontal bar in every plot depicts the mean (±SD) values of the respective parameter measured during a gait cycle.

Independently of the use of an ERB or not, comparing exercises performed with the slow and fast velocities did not show any significant differences (*p* = 0.987) in the peak HJCF. However, consistent with our assumption, the slow velocity trials showed a significantly higher (*p* < 0.001) FTI than those of the fast velocity trials (Figure 7). This was true for all exercises and execution variants. We only found a significant interaction (*p* = 0.009) between ERB and the speed for peak HJCF when performing hip extension exercises. ERB forces were not significantly different between exercises performed with different velocities (refer to the Supplementary Figure 4 in the Supplementary Material).

### Hypothesis 3: Peak and Total Muscle Forces but not Peak Hip JCF of the Movement Leg Will Be Higher Compared to Those During Walking

In all exercises, the peak muscle forces in the movement leg were significantly lower (*p* < 0.05) compared with the respective peak values during walking (Figure 7).The total required muscle forces, i.e., FTI, of each corresponding muscle group of the respective exercise were significantly higher (*p* < 0.05) compared to the same muscle group during the gait trials for all exercises except the hip abduction exercise performed with the fast velocity. Compared to walking, the peak HJCFs were significantly lower (*p* < 0.001) during the fast- and slow-performed hip extension exercises. The peak HJCFs were also significantly lower (*p* = 0.017) compared to walking in the fast-executed hip flexion exercises without an ERB.

## Discussion

The primary aim of this study was to evaluate the muscle forces and associated loads on the hip joint during ERB exercises and to compare these forces with those observed during walking. In agreement with our first hypothesis, both, the stiffer and the softer ERBs, consistently showed significantly higher muscle forces over most of the exercises when compared with those found during exercises performed without an ERB. This outcome confirmed the general assumption that an increase in the training load due to the ERB would lead to higher muscle forces of the targeted musculature. However, comparing muscle forces between the softer and stiffer ERBs did not show a significant difference. HJCF analyses showed a similar trend, with no significant differences in HJCF between the softer and stiffer ERBs. These findings were surprising and partly contradicted our first hypothesis. Comparing the two execution velocities showed, contrary to our second hypothesis, that the variance in velocity does not change the HJCF. However, the required total muscle forces (FTI) were consistently lower during the exercises performed with the fast compared to those with the slow velocity, partly confirming our second hypothesis. When comparing the exercises with walking, the peak muscle forces were significantly lower during all exercises, which was in contrast to our third hypothesis. In addition, the peak HJCFs were similar or significantly lower during the exercises compared with that during walking. On the other hand, the required total muscle forces, i.e., FTI, were significantly higher when exercising with an ERB compared to those during walking, which partly confirmed our third hypothesis.

One of the main goals of the study was to not only quantify the HJCFs but also put them into a perspective using a known and understood metric, which, in our case, were the HJCF found during a gait cycle. However, as walking is generally recommended as a form of aerobic exercise to patients with hip pathologies, such as hip OA, this only gives us a rough idea rather than a full spectrum of acceptable HJCF in people with hip OA (Zhang et al., 2008). This begs the question as to what could be considered to be the upper acceptable limit of HJCF of therapeutic, muscle-strengthening exercises. In people with hip pathologies, jogging is generally considered unsuitable due to the high impacts and the resulting HJCF, which are as high as 5.74 body weight at a speed of 6 km/h (Zhang et al., 2008; Giarmatzis et al., 2015). Taking this into consideration, the HJCFs observed during the ERB exercises in this study were relatively low and did not exceed the values obtained during walking.

Interestingly, compared to the hip flexion and extension exercises, adding an ERB had a minor impact on the muscle and HJCF during the hip abduction exercises. The ERB was attached to the ankle during the hip flexion and extension trials, whereas during the hip abduction trials, the ERB was attached to the femoral condyles. Different ERB locations lead to different moment arms, which might be the reason why adding an ERB barely changed the muscle forces and HJCF during hip abduction exercises.

Comparing the slow- with the fast-performed exercises did not show any significant differences in HJCF. This highlights that a certain variation in execution velocity does not influence hip joint loading and that the velocity of the exercise execution could be determined based on the preference of a patient. Slow velocities, however, significantly increased the total required muscle forces (i.e., FTI) during the exercises compared with fast velocities. In addition to the longer execution duration, slow execution velocities might lead to an increase in agonist–antagonist coactivation due to increased demand on joint stability and therefore a higher FTI. Our simulation results, however, did not confirm this assumption (refer to the Supplementary Material). From a combined training and joint loading perspective, exercises performed with slow velocities are recommended because less repetition and therefore, fewer loading cycles with peak HJCFs are needed to obtain the same FTI compared with the fast-performed exercises.

The magnitude of the HJCF during walking found in this study (mean peak HJCF 2.7 ± 0.45 BW over all participants) was in agreement with the previous findings using instrumented implants (2.4–2.8 BW) but slightly lower compared with the previous simulation studies (3.7–4.9 BW) (Bergmann et al., 1993, 2001; Valente et al., 2013; Modenese et al., 2018; Passmore et al., 2018; Kainz et al., 2020). Different walking velocities, biomechanical models, computational approaches, and study population might be the reason for the observed difference in HJCF between this study and the findings from the previously published simulation studies (Giarmatzis et al., 2015; Kainz et al., 2016; Trinler et al., 2019).

The total required muscle force per exercise (i.e., FTI) increased, as expected, together with an increasing time under tension (slow vs. fast movement execution). The FTI was used as an approximation for muscle work and, although the parameter does not represent the true muscle work, it does give insight into the force profile of a given exercise. Hence, the combination of HJCF, peak muscle forces, and FTI could be used as parameters of exercise control and training design. Furthermore, the ERB type should be chosen to fit the hip range of motion of a patient, as well as to fit the current strength level. The stiffer the ERB, the lesser the range of motion is required to produce the same force. Hence, people with a limited range of motion would potentially benefit from a stiffer ERB to achieve adequate training.

This study included the following limitations. First, we only investigated the impact of two types of ERBs on muscle forces and hip joint loading. The chosen ERBs are often used during rehabilitation exercises but only slightly differed in their force, elongation characteristics. Using different ERBs with larger differences in their force, elongation characteristics (e.g., yellow vs. black ERB from the brand Theraband) would probably lead to more significant differences between the ERBs. Second, greater differences in execution velocities between our slow- and fast-performed trials could lead to different results. These velocities were, however, chosen intentionally as they represent realistic velocities used during rehabilitation exercises. Third, our participants were healthy adults without any known hip pathologies. A different study cohort, e.g., people with hip OA, could perform the exercises with slightly different hip kinematics, which would affect the obtained muscle forces and hip joint loading (Wesseling et al., 2015; Higgs et al., 2019; Diamond et al., 2020). We, however, expect that the relative results, e.g., HJCF due to exercise performed with vs. without an ERB, would be similar to a different study cohort. Fourth, different models and computational approaches might lead to slightly different results (Pieri et al., 2018; Hoang et al., 2019). Fifth, in our ERBs, the relationship between force and elongation was not perfectly linear (Figure 2). Assuming a non-linear relationship and fitting a curve, i.e., second-degree polynomial curve, to our experimental data would have led to a better fit but this would not have affected our findings or conclusion (refer to the Supplementary Material). We chose a linear relationship to be consistent with the previous publications (Hughes et al., 1999). Sixth, considering that a standard gait cycle usually takes around 1 s and our exercise trials took 2 and 3 s for the slow and fast movement executions, respectively, our FTI comparison between the exercises and walking should be interpreted with caution.

## Conclusion

This study highlighted the impact of hip exercises with an ERB on the targeted muscle forces and HJCF. The type of ERB used and the exercise execution velocity had a minor impact on the peak muscle forces and HJCF. Execution velocity, however, does affect the total muscle force required for an exercise. Performing hip exercises without an ERB resulted in similar or lower peak HJCF and lower muscle forces than those found during walking. Adding an ERB during hip exercises increases the peak muscle and HJCF but the values remained below those found during walking. The total muscle forces, i.e., FTI, during hip exercises exceeded the values obtained during walking. This study showed the impact of rehabilitative hip exercises on hip joint loading and the surrounding muscle forces.

## Data Availability Statement

The raw data supporting the conclusions of this article will be made available by the authors, without undue reservation.

## Ethics Statement

The studies involving human participants were reviewed and approved by Ethics Committee of the University of Vienna (00579). The patients/participants provided their written informed consent to participate in this study.

## Author Contributions

CB and HK conceived the original idea and wrote the paper. CB collected the data and prepared the data for the simulations. WK performed the simulations. WK and FD processed the data. WK, HK, and CB performed statistical data analysis. HK supervised the project.

## Conflict of Interest

The authors declare that the research was conducted in the absence of any commercial or financial relationships that could be construed as a potential conflict of interest.

## Publisher's Note

All claims expressed in this article are solely those of the authors and do not necessarily represent those of their affiliated organizations, or those of the publisher, the editors and the reviewers. Any product that may be evaluated in this article, or claim that may be made by its manufacturer, is not guaranteed or endorsed by the publisher.
