# Supplementary material for: Quantifying Muscle Forces and Joint Loading During Hip Exercises Performed With and Without an Elastic Resistance Band
Source: Front Sports Act Living. 2021 Aug 23;3:695383. doi: 10.3389/fspor.2021.695383 (PMC8419330; doi:10.3389/fspor.2021.695383)
Supplement: Supplementary file 1 [file Data_Sheet_1.docx]

SUPPLEMENTARY MATERIAL

## Validation of our simulations

Mean HJCF waveforms of the respective exercises without the ERB across all participants for the movement leg and the stance (supporting) leg were comparable to those obtained from a participant with an instrumented hip implant from the Orthoload database (Figure S1). In all exercises without the ERB, HJCF were higher in the supporting leg compared to the movement leg in our participants, which was in agreement with the participant from the Orthoload database. The shape and maximum values of the Othoload waveforms were similar to the waveforms obtained from our participants, with exception of the HJCF of the movement leg for the hip abduction exercise, which showed higher values in our participants.

Visual comparison between the HJCF from the participants in our study with those found on the Orthoload database showed a reasonable agreement for all exercises (Figure 3) (Bergmann, 2008). Nevertheless, some differences were evident between the HJCF waveforms, especially for the hip abduction exercise. It should be noted that all the HJCF waveforms from the Orthoload database were from one single participant. Differences in HJCF between our results and the values from Orthoload might be caused by a combination of differences in hip kinematics and movement execution velocities, additionally to the different methods to obtain the HJCF (simulations versus in-vivo measurement).


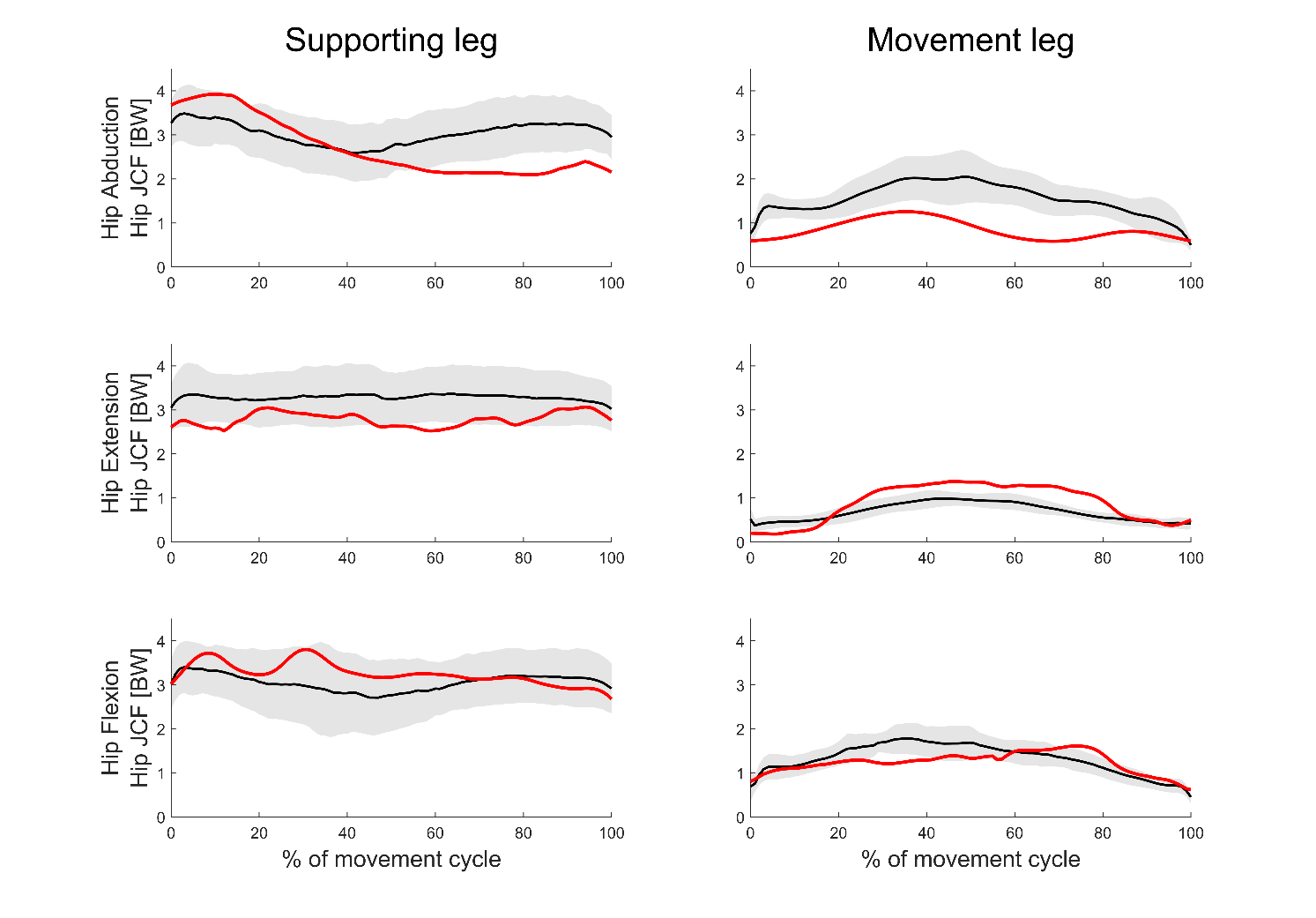


Figure S1. HJCF obtained with the instrumented implant (red waveforms, obtained from participant ‘ebl’ from the Orthoload database) and the mean (±SD) waveforms from our participant (black waveforms and grey shaded areas) for the three exercises performed without the elastic resistance band.

## Additional figures/tables


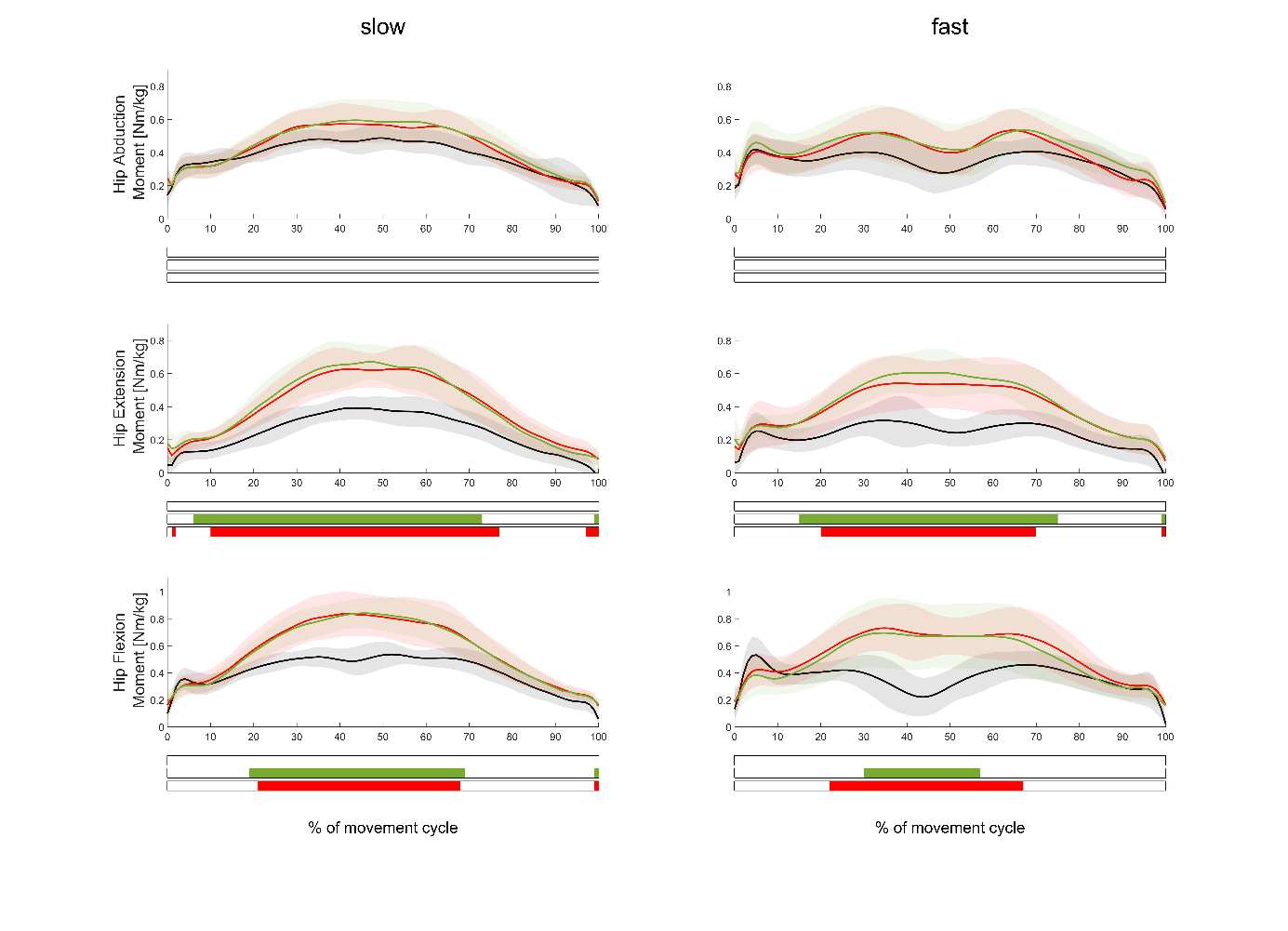


Figure S2. Mean (±SD) hip moment waveforms measured in the executing (movement) leg during hip abduction (top), extension (middle) and flexion (bottom) exercises, as well as during slow (left subplots) and fast (right subplots) velocity. Green, red and black waveforms represent the green (stiffer), red (softer) and no ERB, respectively. Colored bars beneath each plot indicate significant differences between waveforms, whereas the green, red and bue bars represent significant differences between the green versus no ERB, red versus no ERB and green versus red ERB, respectively.


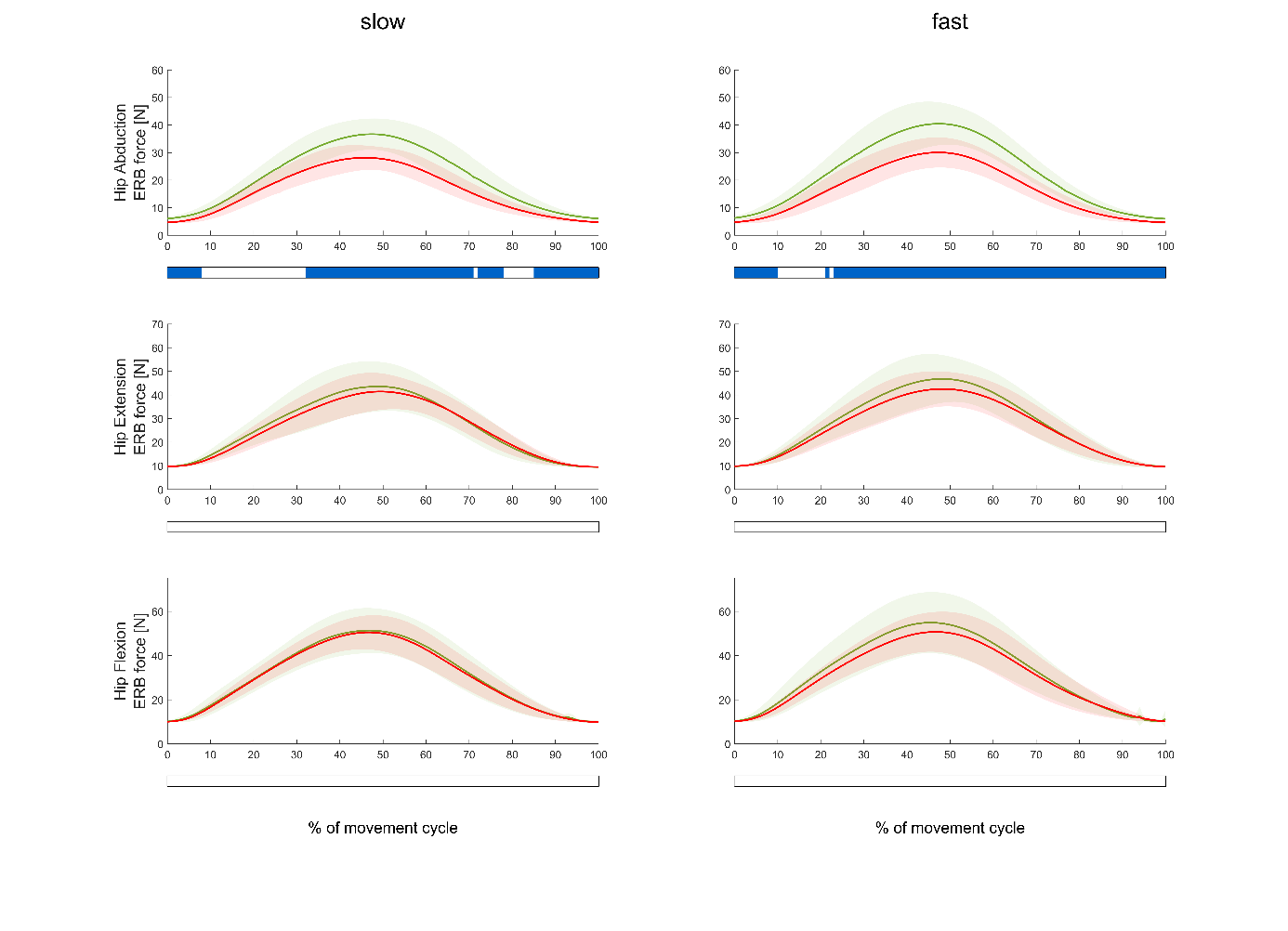


Figure S3. Mean (±SD) ERB forces during slow (left) and fast trials (right) measured in the red (red waveform) and green (red waveform) ERB during abduction (top), extension (middle) and flexion (bottom) exercises. Blue bars beneath each plot indicate significant differences between the forces of the red and green ERB.


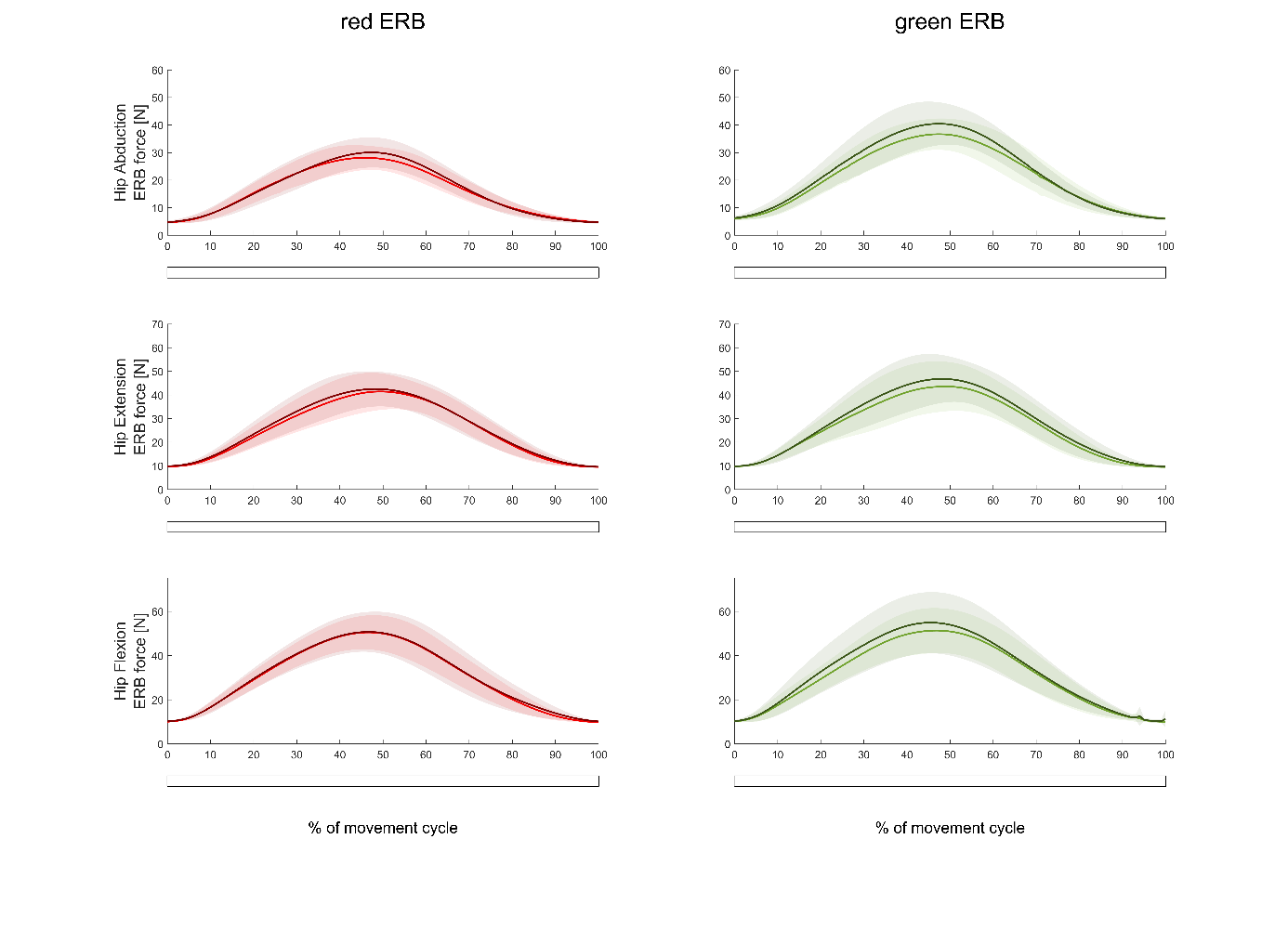


Figure S4. Mean (±SD) ERB forces during fast (dark red and dark green waveforms) and slow trials (light red and light green waveforms) measured in the red (left subplots) and green (right subplots) ERB during abduction (top), extension (middle) and flexion (bottom) exercises. ERB forces were not significantly different between the fast and slow movement executions.


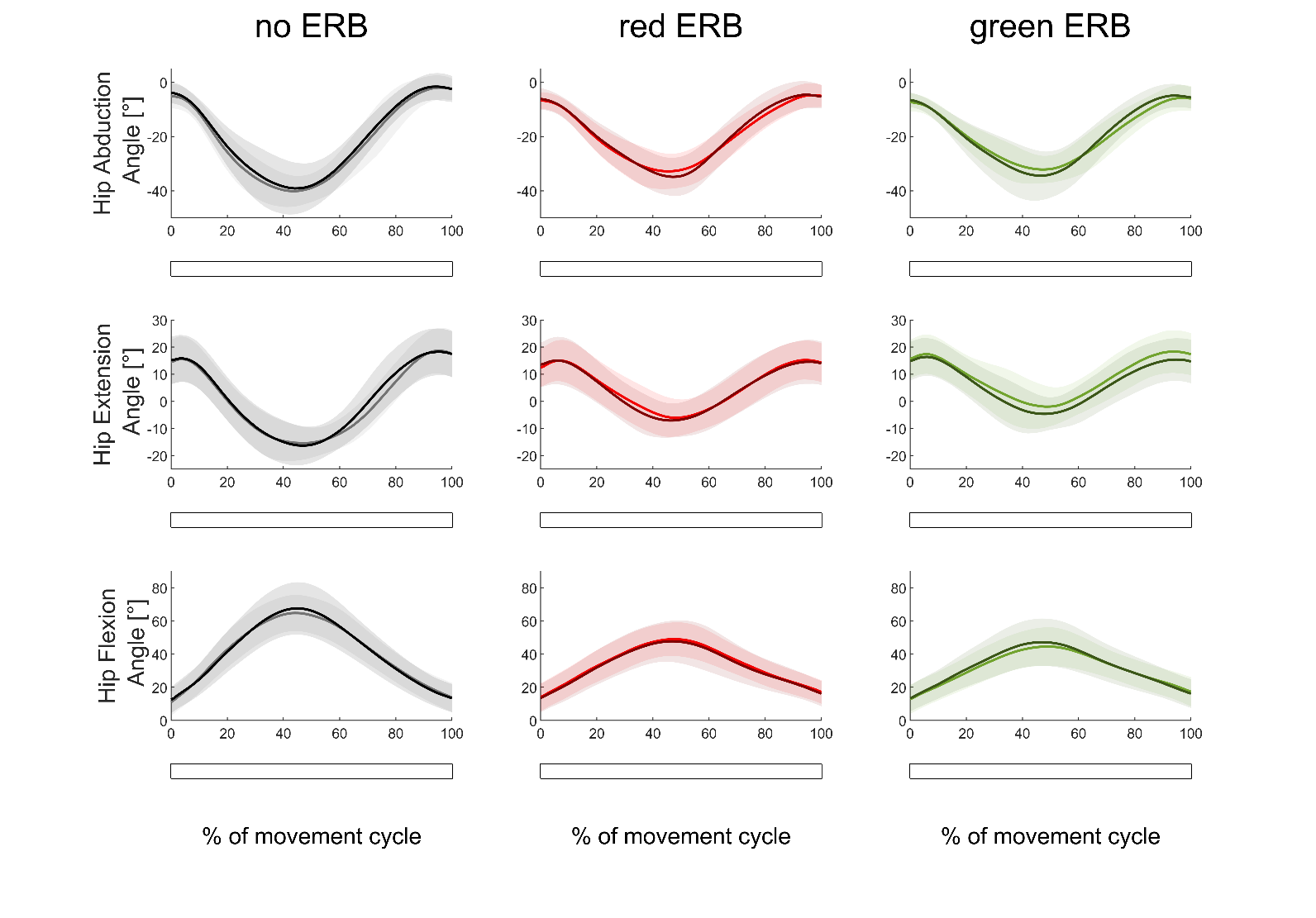


Figure S5. Mean (±SD) hip angle during fast (black, dark red and dark green waveforms) and slow trials (grey, light red and light green waveforms) measured in the trials without (left subplots), with red (middle subplots) and green (right subplots) ERB during abduction (top), extension (middle) and flexion (bottom) exercises. Kinematics were not significantly different between the fast and slow movement executions.


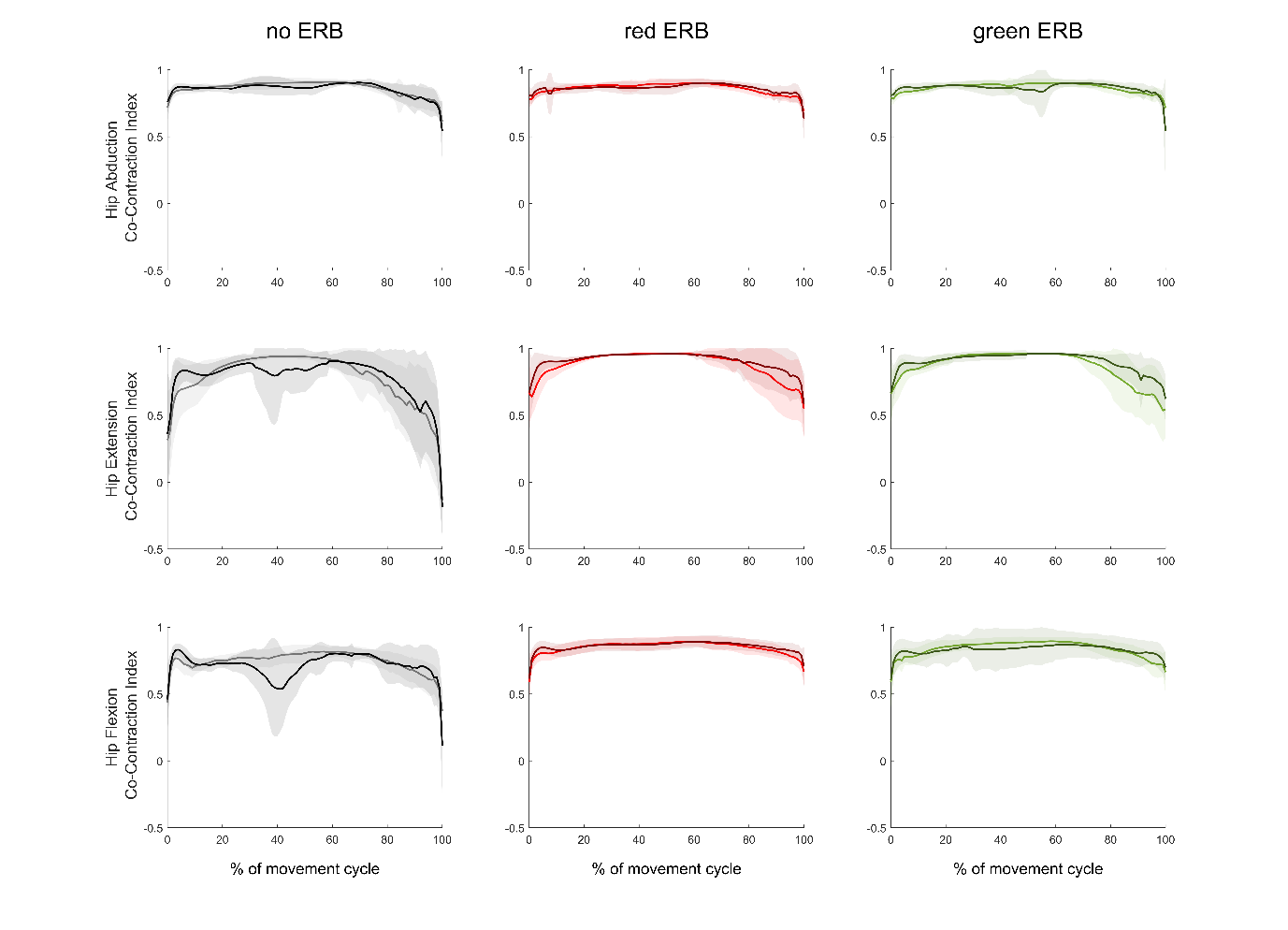


Figure S6. Co-contraction index calculated for the slow (bright waveforms) and fast (dark waveforms) movement.

## Linear versus polynomial fitted curve to model the ERB force production

In our ERBs the relationship between force and elongation was not perfectly linear (Figure S7). Assuming a non-linear relationship and fitting a curve, i.e. 2^nd^ degree polynomial curve, to our experimental data would have led to a better fit but this would not have affected our findings or conclusion (Figure S8 and S9). We chose a linear relationship to be consistent with previous publications (Hughes et al., 1999).


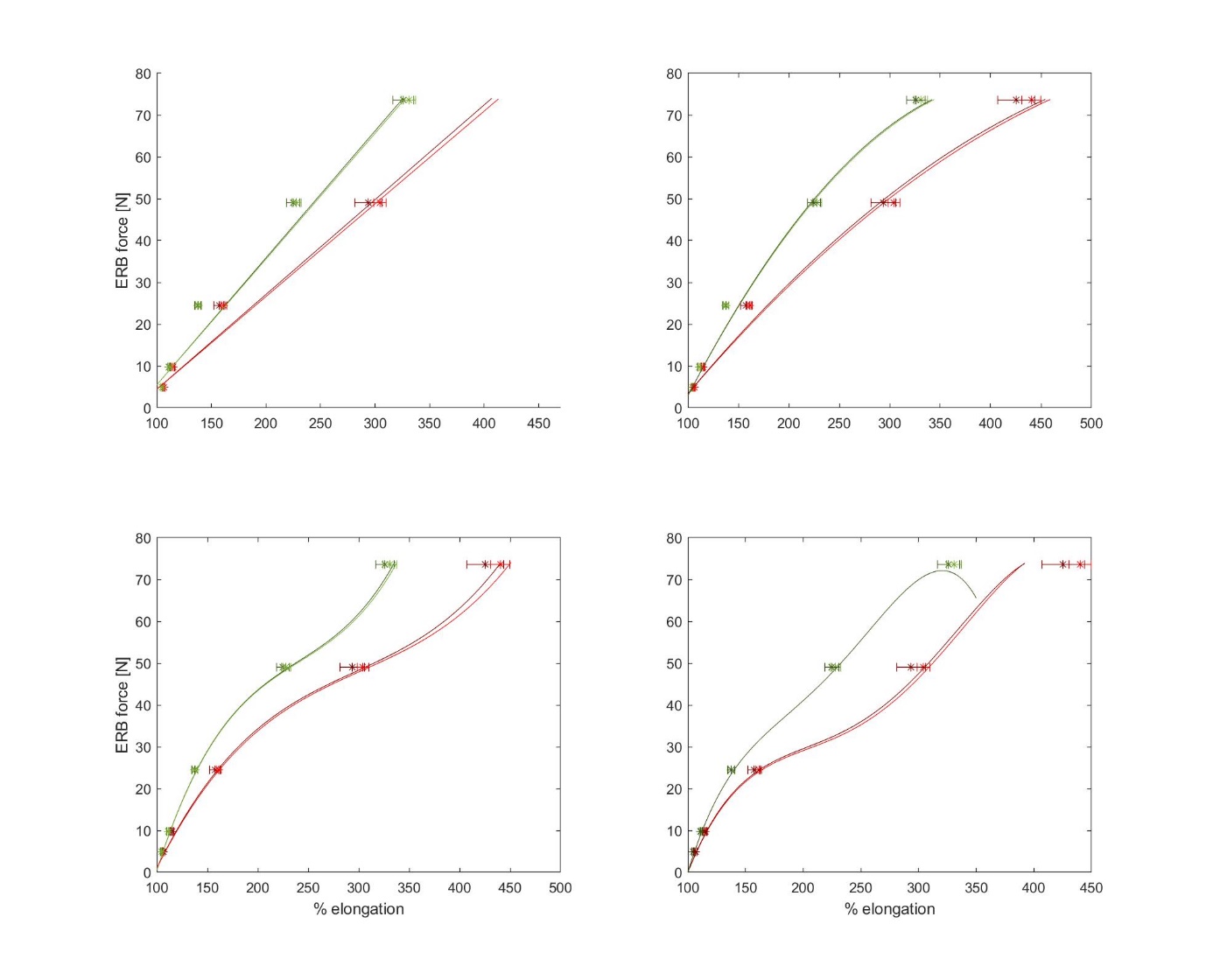


Figure S7. Mean force-elongation curves based on the assumption of a linear relationship (left top plot) and non-linear relationship. Right top plot: 2^nd^ degree polynomial curve, Button left plot: 3^rd^ degree polynomial curve, Button right plot: 4^th^ degree polynomial curve.


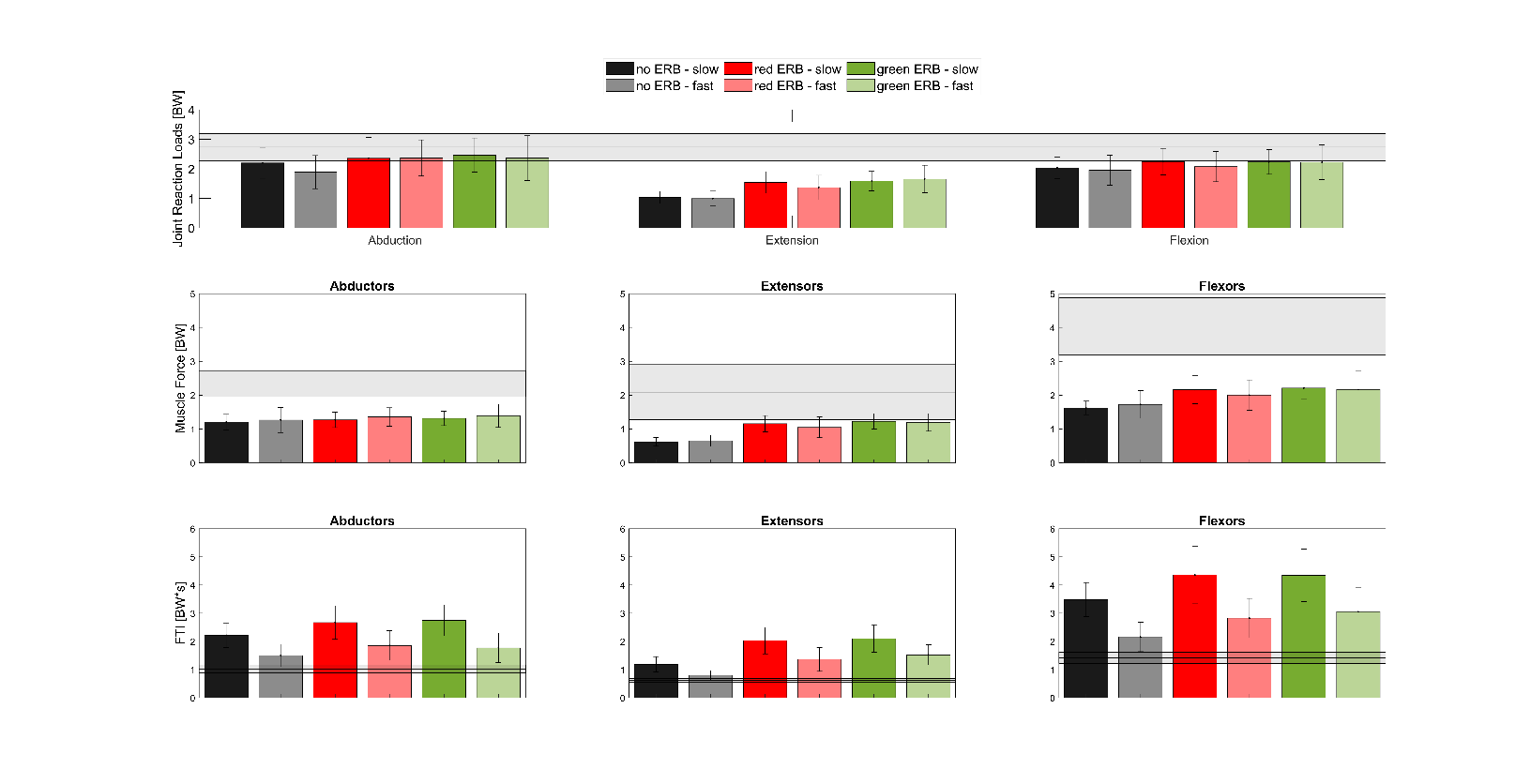


Figure S8. Results based on the assumption of a linear relationship between force and elongation of the ERB.


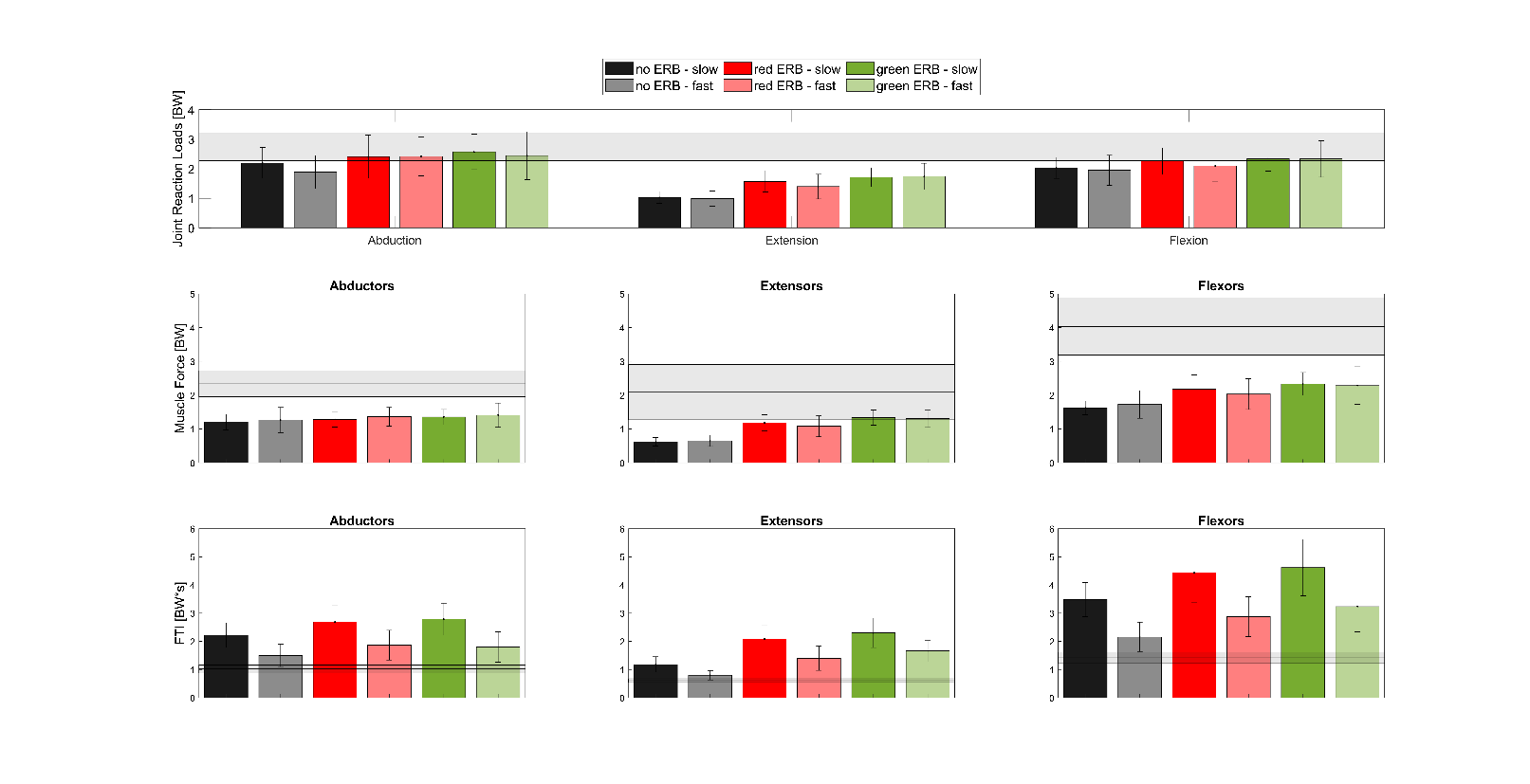


Figure S9. Results based on the assumption of a non-linear (2^nd^ degree polynominal) relationship between force and elongation of the ERB.

Table S1. Peak HJCF and total muscle forces (FTI) during slow and fast execution velocity. The p-values are the result of the statistical comparison between slow and fast parameters. In all analyses the significance level was set to α = 0.05.

| **Peak HJCF** | | | | | | | | |
| --- | --- | --- | --- | --- | --- | --- | --- | --- |
|  |  | **Slow** | | | **Fast** | | |  |
|  |  | **N** | **Mean [%BW]** | **SD [%BW]** | **N** | **Mean [%BW]** | **SD [%BW]** | **p-value** |
| **Abduction** | Without | 7 | 2.02 | 0.53 | 7 | 1.92 | 0.63 |  |
|  | Red | 7 | 2.27 | 0.36 | 7 | 2.35 | 0.61 | 0.987 |
|  | Green | 7 | 2.39 | 0.54 | 7 | 2.41 | 0.59 |  |
| **Flexion** | Without | 11 | 2.01 | 0.38 | 11 | 2.06 | 0.41 |  |
|  | Red | 11 | 2.25 | 0.50 | 11 | 2.13 | 0.55 | 0.676 |
|  | Green | 11 | 2.21 | 0.44 | 11 | 2.19 | 0.64 |  |
| **Extension** | Without | 10 | 1.06 | 0.21 | 10 | 0.95 | 0.24 |  |
|  | Red | 10 | 1.52 | 0.38 | 10 | 1.46 | 0.26 | 0.878 |
|  | Green | 10 | 1.61 | 0.30 | 10 | 1.81 | 0.40 |  |
| **Force-Time Integral (FTI)** | | | | | | | | |
|  |  | **Slow** | | | **Fast** | | |  |
|  |  | **N** | **Mean [BWs]** | **SD [BWs]** | **N** | **Mean [BWs]** | **SD [BWs]** | **p-value** |
| **Abduction** | Without | 7 | 2.27 | 0.50 | 7 | 1.58 | 0.37 | <0.001 |
|  | Red | 7 | 2.89 | 0.54 | 7 | 1.90 | 0.50 | <0.001 |
|  | Green | 7 | 2.94 | 0.51 | 7 | 1.96 | 0.40 | <0.001 |
| **Flexion** | Without | 11 | 3.53 | 0.60 | 11 | 2.27 | 0.42 | <0.001 |
|  | Red | 11 | 4.30 | 1.08 | 11 | 2.88 | 0.73 | <0.001 |
|  | Green | 11 | 4.23 | 0.95 | 11 | 3.00 | 0.92 | <0.001 |
| **Extension** | Without | 10 | 1.26 | 0.28 | 10 | 0.79 | 0.15 | <0.001 |
|  | Red | 10 | 2.05 | 0.41 | 10 | 1.48 | 0.23 | <0.001 |
|  | Green | 10 | 2.25 | 0.37 | 10 | 1.69 | 0.25 | <0.001 |

Table S2. Peak muscle forces and force-time integral (FTI) of each respective muscle group as well as the peak HJCF measured during each exercise variant and during walking. The p-values are the result of the statistical comparison between the respective parameters measured during the exercise and walking trials. In all analyses the significance level was set to α = 0.05. n.s.=not significant different; WB=without band; R=red ERB; G=green ERB

|  |  | **Peak Muscle Forces** | | | | | **Force-Time Integral (FTI)** | | | | **Peak HJCF** | | | |
| --- | --- | --- | --- | --- | --- | --- | --- | --- | --- | --- | --- | --- | --- | --- |
|  |  |  | **N** | **Mean [%BW]** | **SD [%BW]** | **p-values** | **N** | **Mean [BWs]** | **SD [BWs]** | **p-values** | **N** | **Mean [%BW]** | **SD [%BW]** | **p-value** |
| **Gait** | **Abd** | | 16 | 2.34 | 0.39 | - | 16 | 1.02 | 0.14 | - | 16 | 2.73 | 0.46 | - |
|  | **Flex** | | 16 | 4.03 | 0.85 | - | 16 | 1.42 | 0.20 | - |  |  |  |  |
|  | **Ext** | | 16 | 2.09 | 0.82 | - | 16 | 0.61 | 0.07 | - |  |  |  |  |
| **Abd** | slow | **WB** | 10 | 1.21 | 0.24 | **<0.01** | 10 | 2.22 | 0.46 | **<0.001** | 10 | 2.20 | 0.55 | n.s. |
|  | fast |  | 8 | 1.25 | 0.42 | **<0.01** | 8 | 1.48 | 0.44 | n.s. | 8 | 1.85 | 0.62 | n.s. |
|  | slow | **R** | 10 | 1.30 | 0.18 | **<0.01** | 10 | 2.82 | 0.46 | **<0.001** | 10 | 2.49 | 0.67 | n.s. |
|  | fast |  | 8 | 1.33 | 0.36 | **<0.01** | 8 | 1.74 | 0.64 | n.s. | 8 | 2.19 | 0.72 | n.s. |
|  | slow | **G** | 10 | 1.34 | 0.24 | **<0.01** | 10 | 2.88 | 0.44 | **<0.001** | 10 | 2.54 | 0.61 | n.s. |
|  | fast |  | 8 | 1.33 | 0.44 | **<0.01** | 8 | 1.76 | 0.68 | n.s. | 8 | 2.18 | 0.84 | n.s. |
| **Flex** | slow | **WB** | 11 | 1.62 | 0.21 | **<0.001** | 11 | 3.53 | 0.60 | **<0.001** | 11 | 2.01 | 0.39 | n.s. |
|  | fast |  | 12 | 1.84 | 0.30 | **<0.001** | 12 | 2.24 | 0.41 | **<0.01** | 12 | 2.07 | 0.39 | **0.017** |
|  | slow | **R** | 11 | 2.13 | 0.44 | **<0.001** | 11 | 4.30 | 1.08 | **<0.001** | 11 | 2.25 | 0.50 | n.s. |
|  | fast |  | 12 | 1.96 | 0.49 | **<0.001** | 12 | 2.79 | 0.76 | **<0.01** | 12 | 2.06 | 0.57 | n.s. |
|  | slow | **G** | 11 | 2.15 | 0.30 | **<0.001** | 11 | 4.23 | 0.95 | **<0.001** | 11 | 2.21 | 0.44 | n.s. |
|  | fast |  | 12 | 2.14 | 0.56 | **<0.001** | 12 | 3.01 | 0.88 | **<0.01** | 12 | 2.21 | 0.62 | n.s. |
| **Ext** | slow | **WB** | 12 | 0.62 | 0.13 | **<0.05** | 12 | 1.20 | 0.29 | **<0.001** | 12 | 1.04 | 0.20 | **<0.001** |
|  | fast |  | 12 | 0.66 | 0.17 | **<0.05** | 12 | 0.79 | 0.15 | **<0.05** | 12 | 0.96 | 0.25 | **<0.001** |
|  | slow | **R** | 12 | 1.13 | 0.22 | **<0.05** | 12 | 1.97 | 0.43 | **<0.001** | 12 | 1.52 | 0.35 | **<0.001** |
|  | fast |  | 12 | 1.05 | 0.26 | **<0.05** | 12 | 1.37 | 0.36 | **<0.05** | 12 | 1.37 | 0.37 | **<0.001** |
|  | slow | **G** | 12 | 1.23 | 0.24 | **<0.05** | 12 | 2.11 | 0.51 | **<0.001** | 12 | 1.58 | 0.34 | **<0.001** |
|  | fast |  | 12 | 1.22 | 0.28 | **<0.05** | 12 | 1.57 | 0.36 | **<0.05** | 12 | 1.66 | 0.50 | **<0.001** |

## Detailed results from the SPSS analyses

1. **Second hypothesis** - peak HJCF and the force-time integral (FTI) were compared between the slow and fast exercise executions.

**Peak HJCF**

Abd

F(1, 6) = 0.00, p = 0.987, partial eta squared = 0.00

No interaction band versus speed

Flex

F(1, 10) = 1.86, p = 0.676, partial eta squared = 0.018

No interaction band versus speed

Ext

F(1, 9) = 0.025, p = 0.878, partial eta squared = 0.003

Signif interaction band and speed F(2, 18) = 6.173, p=0.009, partial eta squared = 0.407

**FTI**

Abd

F(1, 6) = 65.94, p < 0.001, partial eta squared = 0.917

No interaction band versus speed

Flex

F(1, 10) = 126.99, p < 0.001, partial eta squared = 0.927

No interaction band versus speed

Ext

F(1, 9) = 84.572, p < 0.001, partial eta squared = 0.904

No interaction band versus speed

Post-hoc comparison p < 0.001 for all three comparisons

1. **Third hypothesis -** peak and total muscle forces but not peak HJCF of the executing leg will be higher compared to walking

**Peak HJCF**

Abd slow

F(1.3, 11.7) = 1.644, p = 0.231, partial eta squared = 0.154

Abd fast

F(3, 21) = 4.396, p = 0.015, partial eta squared = 0.386

Contrast F(1, 7) = 5.287, p=0.055 -> no significant post-hoc results

Flex slow

F(3, 30) = 6.058, p = 0.002, partial eta squared = 0.377

Contrast F(1, 10) =8.666, p = 0.015

Post hoc -> no signif difference

Flex fast

F(3, 33) = 5.031, p = 0.006, partial eta squared = 0.314

Contrast F(1, 11) =10.53, p = 0.008

Post hoc -> gait vs ohne fast p=0.017 (flex fast signif lower compared to walking)

Ext slow

F(1.7, 18.3) = 57.892, p < 0.001, partial eta squared = 0.84

Contrast F(1, 11) =70.462, p < 0.001

Post hoc -> gait vs all ext trials p < 0.001 (gait signif higher)

Ext fast

F(3, 33) = 56.744, p < 0.001, partial eta squared = 0.838

Contrast F(1, 11) =127.339, p < 0.001

Post hoc -> gait vs all ext trials p < 0.001 (gait signif higher)

**Peak muscle force**

Abd slow

F(1.3, 11.7) = 34.919, p <0.001, partial eta squared = 0.795

Contrast F(1, 9) = 40.999, p<0,001

Post hoc -> gait vs all abd trials p < 0.01 (gait signif higher)

Abd fast

F(3, 21) = 24.949, p <0.001, partial eta squared = 0.781

Contrast F(1, 7) = 46.104, p<0,001

Post hoc -> gait vs all abd trials p < 0.01 (gait signif higher)

Flex slow

F(1.29, 12.923) = 49.285, p < 0.001, partial eta squared = 0.831

Contrast F(1, 10) =54.129, p <0.001

Post hoc -> gait vs all flex trials p < 0.001 (gait signif higher)

Flex fast

F(3, 33) = 35.226, p < 0.001, partial eta squared = 0.762

Contrast F(1, 11) =51.701, p < 0.001

Post hoc -> gait vs all flex trials p < 0.001 (gait signif higher)

Ext slow

F(1.12, 12.32) = 29.325, p < 0.001, partial eta squared = 0.727

Contrast F(1, 11) =25.675, p < 0.001

Post hoc -> gait vs all ext trials p < 0.05 (gait signif higher)

No band p < 0.001; red p = 0.005; green p = 0.013

Ext fast

F(1.29, 14.22) = 24.575, p < 0.001, partial eta squared = 0.691

Contrast F(1, 11) =24.578, p < 0.001, partial eta squared = 0.691

Post hoc -> gait vs all ext trials p < 0.05 (gait signif higher)

No band p < 0.001; red p = 0.007; green p = 0.010

**FTI**

Abd slow

F(3, 27) = 73.480, p <0.001, partial eta squared = 0.891

Contrast F(1, 9) = 111.854, p<0,001, partial eta squared = 0.926

Post hoc -> gait vs all abd trials p < 0.001 (gait signif lower)

Abd fast

F(3, 21) = 7.071, p = 0.002, partial eta squared = 0.503

Contrast F(1, 7) = 11.447, p=0.012, partial eta squared = 0.621

Post hoc -> no signif. difference

Flex slow

F(3, 30) = 51.567, p < 0.001, partial eta squared = 0.838

Contrast F(1, 10) =95.359, p <0.001, partial eta squared = 0.905

Post hoc -> gait vs all flex trials p < 0.001 (gait signif higher)

Flex fast

F(2.074, 22.817) = 21.138, p < 0.001, partial eta squared = 0.658

Contrast F(1, 11) =35.490, p < 0.001, partial eta squared = 0.763

Post hoc -> gait vs all flex trials p < 0.01 (gait signif lower)

Ext slow

F(3, 33) = 89.323, p < 0.001, partial eta squared = 0.890

Contrast F(1, 11) =124.815, p < 0.001, partial eta squared = 0.919

Post hoc -> gait vs all ext trials p < 0.001 (gait signif lower)

Ext fast

F(3, 33) = 46.458, p < 0.001, partial eta squared = 0.809

Contrast F(1, 11) =76.351, p < 0.001, partial eta squared = 0.874

Post hoc -> gait vs all ext trials p < 0.05 (gait signif lower)

No band p = 0.047; red p < 0.001; green p < 0.001
